# SHAP enhanced transformer GWO boosting model for transparent and robust anomaly detection in IIoT environments

**DOI:** 10.1038/s41598-025-25033-0

**Published:** 2025-11-20

**Authors:** Mohammed Aly, Naif M. Alotaibi

**Affiliations:** 1 Department of Artificial Intelligence, Faculty of Artificial Intelligence, Egyptian Russian University, Badr City, 11829 Egypt; 2 Department of Computer Science, College of Science and Humanities Dawadmi, Shaqra University, Shaqra, 11961 Saudi Arabia

**Keywords:** Anomaly detection, Industrial internet of things (IIoT), Transformer networks, Grey wolf optimizer (GWO), Boosting classifier, Explainable artificial intelligence (XAI), SHAP (SHapley additive exPlanations), Scalability, Smart factories, SCADA/IoT dashboards, Engineering, Mathematics and computing

## Abstract

The rapid adoption of the Industrial Internet of Things (IIoT) has transformed factory operations by enabling real-time monitoring and automation, but it has also exposed production environments to frequent anomalies and cyber-physical risks. Traditional machine learning approaches such as Random Forests, Support Vector Machines, and ensemble boosting methods have demonstrated strong performance, yet they often face limitations when dealing with data imbalance, temporal dependencies, and concept drift in evolving sensor streams. In this study, we propose a hybrid framework that integrates a temporal transformer encoder with a Logistic Boosting classifier, enhanced through bio-inspired feature optimization using the Grey Wolf Optimizer. The transformer component captures sequential patterns in sensor data, while the optimization layer refines feature selection to improve generalization. Logistic Boosting then provides robust classification, balancing sensitivity and precision under imbalanced conditions. Experiments were conducted on a real-world six-month dataset of 15,000 sensor readings collected from a smart manufacturing facility. The proposed model achieved an accuracy of 98.2%, with 96.7% precision, 97.1% recall, an F1-score of 0.969, and an AUC of 0.996, outperforming the baseline Logistic Boosting model (96.6% accuracy, AUC 0.992). In addition to superior predictive performance, the framework demonstrated resilience under data drift scenarios and maintained low inference latency suitable for edge deployment. In addition to high predictive accuracy, the framework provides explainable outputs using SHAP analysis, ensuring that anomaly alerts are transparent and interpretable for industrial operators. These findings highlight the effectiveness of combining temporal transformers, boosting ensembles, and metaheuristic optimization for accurate detection of unusual events in IoT-enabled factories, offering a framework that can be applied across different factories or scaled to larger datasets without major redesign towards secure and adaptive industrial systems.

## Introduction

 The convergence of industrial automation and the Internet of Things (IoT) has given rise to the Industrial Internet of Things (IIoT), which is now central to modern smart manufacturing. By connecting sensors, actuators, and cyber-physical systems, IIoT enables continuous monitoring, predictive maintenance, and operational efficiency. At the same time, this connectivity introduces risks: unusual system behaviors—whether from equipment faults, environmental disturbances, or cyberattacks—can threaten production, safety, and data integrity. Accurately detecting such anomalies in real time is therefore a key challenge for building trustworthy and resilient industrial systems^1,2^.

Classical machine learning methods such as Random Forests, Support Vector Machines, and boosting ensembles have shown promise for identifying unusual behaviors in sensor data that may indicate equipment faults or cyberattacks, in IIoT environments, especially in handling heterogeneous sensor data and class imbalance^3–5^. Yet, these approaches often fail to capture complex temporal dependencies and degrade under data drift or evolving operating regimes^6^. Deep learning methods such as CNNs and LSTMs address sequential dependencies^7^ but face challenges related to computational cost, training requirements, and interpretability^8^. More recently, transformer-based models have been adapted for IIoT anomaly detection, offering superior capacity to model long-range dependencies in sensor data^9^. However, transformers can suffer from feature redundancy and remain difficult to deploy efficiently on edge devices.

To mitigate these issues, hybrid approaches that combine deep temporal modeling, optimization, and ensemble learning have attracted increasing attention. Bio-inspired optimization techniques such as Grey Wolf Optimizer and Particle Swarm Optimization have proven effective for feature selection and model tuning in IoT contexts^10,11^, while adaptive models are increasingly emphasized for handling data drift^12^. Recent advances in anomaly detection for IIoT have increasingly focused on hybrid models that integrate deep learning, optimization, and interpretability. For example, Chen et al.^13^ demonstrated that combining explainable deep learning with feature selection strategies can enhance intrusion detection accuracy while maintaining transparency. Similarly, Albalwy and Almohaimeed^14^ integrated multiple feature selection approaches with deep models for real-time intrusion detection in AIoT environments, highlighting the growing demand for interpretable yet efficient detection frameworks. These efforts underscore the importance of unifying predictive accuracy, interpretability, and efficiency, which motivates the design of our Transformer–GWO–Boosting (TGB) model.

In this study, we propose the Transformer–GWO–Boosting (TGB) framework: a hybrid anomaly detection model that integrates (i) a lightweight temporal transformer encoder for long-range dependency modeling, (ii) GWO-based feature selection to improve drift robustness and reduce redundancy, (iii) Logistic Boosting for high recall and SHAP-based interpretability, and (iv) A deployment-ready hybrid architecture that simultaneously advances accuracy, robustness, interpretability, and efficiency, establishing a new benchmark for anomaly detection in IIoT environments. Using a six-month dataset comprising 15,000 sensor readings from an operational manufacturing facility, we benchmark TGB against state-of-the-art baselines and demonstrate improvements in accuracy, robustness, and adaptability. The framework is explicitly designed with deployment feasibility in mind, establishing a practical and scalable solution for anomaly detection in IIoT-driven factories.

The remainder of this paper is organized as follows: Sect. “Related works” reviews related works, Sect. “Proposed methodology” describes the proposed methodology, Sect. "Experiments and results" presents results, Sect. “Discussion” discusses findings, Sect. "Limitations, deployment workflow, and explainability in iiot applications" addresses limitations and industrial deployment, and Sect. "Conclusion and future work" concludes with future directions.

## Related works

Research on anomaly detection in the Industrial Internet of Things (IIoT) spans classical machine learning, deep temporal modeling, and optimization-driven hybrids, with growing emphasis on deployment under edge and drift conditions. Early approaches rely on tabular learners such as Random Forests, Support Vector Machines, and boosting ensembles, which handle heterogeneous features and class imbalance effectively in factory datasets. Comparative studies confirm that boosting methods remain strong baselines for IIoT anomaly classification, often outperforming tree ensembles and margin-based methods on accuracy and AUC, though they struggle to capture long-range temporal dependencies and may degrade under shifting operating regimes^3,15^.

To better exploit temporal structure, deep learning has moved from recurrent networks to attention-based architectures. Transformers designed for industrial anomaly detection—such as the Inductive Transformer (ITran)—model long-range dependencies and cross-sensor interactions more effectively than CNN/LSTM pipelines, improving detection and localization in complex production lines. Extensions to multivariate sensor streams report gains in robustness and generalization, although compute and memory overheads remain a concern for edge deployment. These developments motivate hybrid designs that retain transformer temporal modeling while delegating final discrimination to efficient learners^16,17^.

Feature quality and computational efficiency have also been improved through metaheuristic optimization. Grey Wolf Optimizer (GWO) and related variants have shown competitive results in high-dimensional classification by balancing exploration and exploitation to reduce redundancy and overfitting before classification^17–19^. Several recent studies combine evolutionary optimization with neural architectures for IIoT anomaly detection. Khan et al.^20^ proposed an adaptive hybrid framework integrating neural networks and genetic algorithms for feature optimization, while Dong et al.^21^ introduced a GA-Att-LSTM for real-time fault detection under edge–cloud collaboration. These works highlight the value of bio-inspired selection for robust anomaly detection. Our study builds on this direction by coupling GWO-based feature refinement with temporal transformers and boosting ensembles.

Deployment constraints further shift attention to lightweight models and drift resilience. Edge-centric studies show that compact learners and efficient deep models can meet latency budgets while maintaining acceptable accuracy, provided that communication and memory overheads are minimized. In parallel, concept-drift literature emphasizes detecting and adapting to distributional shifts through methods such as KSWIN, DDM, and unsupervised adaptation^22–25^. In addition to accuracy and robustness, deployment feasibility has been recognized as a key challenge in IIoT anomaly detection. Sun et al.^26^ demonstrated that memory-efficient anomaly detectors like TinyAD can run effectively on resource-constrained IIoT devices, underscoring the importance of deployment-aware designs. While such approaches focus primarily on efficiency, our work aims to balance accuracy, drift robustness, explainability, and feasibility at the edge.

Comprehensive surveys map this evolving landscape. Reviews of IoT/IIoT anomaly detection catalogue classical ML, ensembles, and deep methods, and highlight open challenges around temporal generalization, imbalance, explainability, and drift-aware evaluation^27,28^. Recent reviews of explainable AI (XAI) in industrial fault diagnosis stress the need for transparent detectors to support safety-critical decision-making^29^. Similarly, Chen et al.^13^ showed that explainable deep learning with feature selection improves intrusion detection, while Albalwy and Almohaimeed^14^ highlighted real-time hybrid frameworks for AIoT environments. These insights align with our focus on unifying predictive accuracy, drift robustness, interpretability, and deployment readiness.

Table 1 compares representative baseline methods for IIoT anomaly detection with respect to imbalance handling, drift adaptation, optimization, and explainability. Classical learners such as Random Forests and SVMs partially address imbalance but lack drift and optimization strategies. Deep models such as LSTM autoencoders and transformers capture temporal dependencies but remain drift-sensitive and provide limited interpretability^22^. Logistic Boosting offers better calibration under imbalance and supports SHAP-based explanations but struggles with drift and redundant features. In contrast, the proposed Transformer–GWO–Boosting (TGB) framework combines imbalance-aware boosting, drift-robust temporal encoding, bio-inspired feature optimization, and operator-oriented interpretability, directly addressing these documented shortcomings.

**Table 1:** Comparison of representative baseline methods for IIoT anomaly detection.

Method	Handles imbalance	Drift adaptation	Optimization applied	Explainability support
Random Forest (RF)	Partial (class weights)	Weak (static trees)	No	Limited (feature importance)
Support Vector Machine (SVM)	Partial (weighted kernels)	Weak (sensitive to distribution shift)	No	Limited (support vectors)
LSTM Autoencoder (LSTM-AE)	No	Moderate (sequence modeling but drift-sensitive)	No	Minimal
ITran (Transformer)	No	Moderate (temporal attention captures context)	No	Limited (attention visualization)
Logistic Boosting (LB)	Yes (instance weighting)	Weak (no drift detection)	No	Strong (tree-based SHAP values)
**Proposed TGB (Transformer + GWO + Boosting)**	**Yes (dynamic weighting)**	**Strong (tested under drift scenarios)**	**Yes (bio-inspired feature selection)**	**Strong (SHAP explanations on boosted features)**

As summarized in Table 1, existing anomaly detection approaches for IIoT exhibit important gaps. Classical learners only partially handle imbalance and fail under distributional shifts, while deep models capture temporal dependencies but remain drift-sensitive and difficult to interpret. Boosting methods provide calibration and feature attribution but struggle with drift and redundancy^3^. These limitations motivate the need for an integrated framework that unifies temporal modeling, feature optimization, and interpretability. In the following section, we present the proposed Transformer–GWO–Boosting (TGB) framework, designed to directly address these challenges in real-world IIoT environments.

Table 2 contrasts representative baseline methods against the proposed Transformer–GWO–Boosting (TGB) model across four dimensions critical for IIoT anomaly detection: predictive accuracy, robustness under drift, explainability, and suitability for edge deployment. While individual methods excel in isolated aspects (e.g., transformers in temporal modeling, boosting in interpretability), none simultaneously address all four. The TGB framework uniquely combines these strengths into a single deployment-ready pipeline.

**Table 2:** Comparative novelty dimensions of existing approaches versus the proposed TGB framework.

Method	Accuracy	Drift robustness	Explainability	Edge deploy ability
Random Forest (RF)	Moderate	Weak	Partial (feature importance only)	Strong (low latency, low memory)
Support Vector Machine (SVM)	Moderate	Weak	Limited (support vectors)	Moderate (higher latency, sensitive to scale)
LSTM Autoencoder (LSTM-AE)	High	Moderate	Minimal	Weak (high memory, slow inference)
Transformer (ITran)	High	Moderate	Limited (attention visualization)	Weak–Moderate (high compute cost)
Logistic Boosting (LB)	High	Weak	Strong (SHAP explanations)	Strong (lightweight, efficient)
**Proposed TGB**	**Highest**	**Strong**	**Strong (feature + temporal SHAP)**	**Strong (balanced accuracy and efficiency)**

## Proposed methodology

The proposed Transformer–GWO–Boosting (TGB) framework integrates temporal transformers, bio-inspired feature selection, and ensemble boosting into a modular pipeline. Its goal is to achieve accurate, interpretable, and deployment-ready anomaly detection for IIoT environments. The design emphasizes three principles: (i) temporal generalization via transformers, (ii) feature redundancy reduction through Grey Wolf Optimizer (GWO), and (iii) recall-oriented boosting with SHAP-based interpretability. The modular architecture facilitates edge deployment and supports incremental retraining when sensors or operating conditions evolve.

### Overview of the pipeline

The proposed Transformer–GWO–Boosting (TGB) framework follows a modular pipeline that integrates preprocessing, temporal encoding, feature refinement, and ensemble classification. Multivariate sensor streams are first preprocessed for temporal alignment and missing values. A lightweight transformer encoder then produces embeddings that capture long-range dependencies and cross-sensor interactions. Grey Wolf Optimizer (GWO) is applied to refine feature subsets, reducing redundancy and improving drift robustness. Finally, a Logistic Boosting ensemble generates anomaly predictions while supporting SHAP-based interpretability. To ensure practical reliability, the framework is trained and tested under temporally stratified splits, with additional drift scenarios introduced to mimic real-world operational changes. Figure 6 provides a workflow overview of this end-to-end process.

### Data preprocessing and temporal framing

The framework ingests five key sensor modalities: ambient temperature, light intensity, motion detection, window/door status, and power consumption. To preserve temporal context, each instance is reframed into overlapping windows of length $\:T$. Missing values are imputed via local median interpolation to prevent biasing rare anomalies, while outliers are mitigated using a modified Z-score filter, consistent with prior baselines. To reduce variance across sensors, each feature $\:x$ is standardized as:

$$\:\stackrel{\sim}{x}=\frac{x-\mu\:}{\sigma\:},$$

where $\:\mu\:$ and $\:\sigma\:$ are running estimates of mean and standard deviation computed per sensor over the training period.

To emulate real-world dynamics, a temporally stratified split is applied: months 1–4 are used for training, month 5 for validation, and month 6 for testing. In addition, synthetic drift scenarios are introduced by perturbing feature distributions (mean shifts and variance scaling), enabling evaluation of robustness to evolving operating conditions.

### Transformer-based temporal encoding

**Fig. 1:**
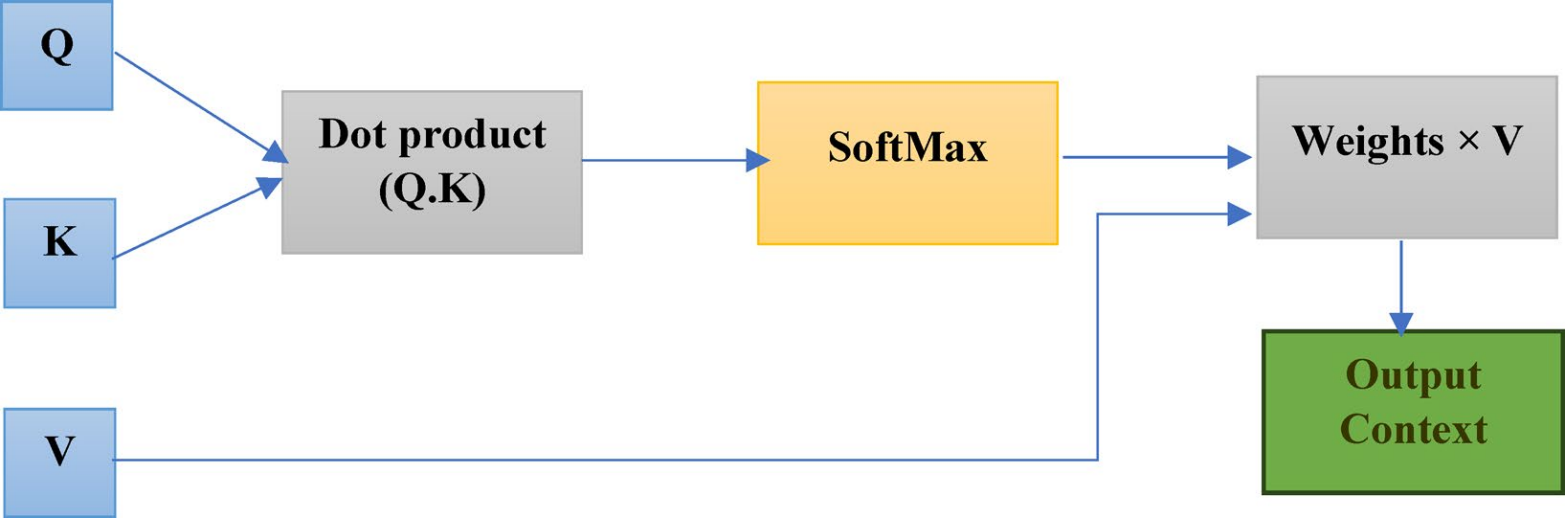
Visualization of the transformer attention mechanism. Queries (Q) and Keys (K) are combined via a dot product and normalized with a softmax function to generate attention weights, which are then applied to Values (V) to compute the final context-aware output representation.

To model long-range dependencies, we adopt a lightweight transformer encoder adapted to multivariate sensor streams. Given an input window $\:X\in\:{\mathbb{R}}^{T\times\:F}$, where $\:T$ is the temporal length and $\:F$ the number of raw features, a shallow temporal convolutional stem first extracts local patterns and reduces sequence length, yielding $\:X{\prime\:}\in\:{\mathbb{R}}^{T{\prime\:}\times\:F}$. Positional encodings are then added to preserve sequential order. Each attention block computes scaled dot-product attention as:

$$\:Attention\left(Q,K,V\right)=softmax\left(\frac{Q{K}^{\top\:}}{\sqrt{{d}_{k}}}\right)V,$$

where queries ($\:Q$), keys ($\:K$), and values ($\:V$) are linear projections of the input, and $\:{d}_{k}$is the key dimension. Residual connections and layer normalization stabilize training across the $\:L$ stacked self-attention layers. The output embeddings are aggregated using attention pooling to produce a fixed-length representation $\:z\in\:{\mathbb{R}}^{D}$ for each window. This design balances representational power with computational efficiency, drawing inspiration from recent transformer adaptations for sensor data^16,30,31^. Figure 1 illustrates the scaled dot-product attention mechanism, highlighting how Q, K, and V interact to yield context-aware representations.

The resulting embeddings are then refined via GWO-based feature selection (see Sect. 3.4).

### Bio-inspired feature selection with grey Wolf optimizer

While transformer embeddings capture temporal dynamics, redundant or noisy raw features can still degrade generalization and increase latency. To address this, the Grey Wolf Optimizer (GWO) is employed as a wrapper-based feature selection method operating on a hybrid input space of transformer-derived embeddings and candidate raw features.

**Fig. 2:**
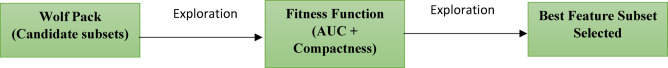
Grey Wolf Optimizer (GWO) feature selection flow. Candidate feature subsets are evaluated by the fitness function, which balances predictive accuracy (AUC) with compactness. The best-performing subset is iteratively refined and selected.

GWO maintains a population of feature subsets and evaluates each using a fitness function that balances predictive accuracy and compactness:

$$\:F\left(S\right)=\alpha\:\cdot\:(1-{AUC}_{val}\left(S\right))+\beta\:\cdot\:\frac{\mid\:S\mid\:}{{F}_{max}}\varvec{}\varvec{},\:$$

where $\:S$ is the feature subset, $\:{AUC}_{val}\left(S\right)$ is the cross-validated validation AUC of the downstream classifier, $\:\mid\:S\mid\:$ is the number of features selected, and $\:\alpha\:,\:\beta\:\:$control the trade-off between accuracy and parsimony. Through its exploration–exploitation dynamics, GWO converges on compact subsets that retain detection performance while reducing inference cost. Preliminary results showed that GWO selected four to five features while preserving over 99% of baseline AUC, effectively eliminating redundancy^16,32^. Figure 2 illustrates the GWO feature selection process, showing how candidate subsets are iteratively evaluated by the fitness function and refined until the optimal set is chosen.

### Logistic boosting classifier and calibration

The final classification stage employs a Logistic Boosting ensemble applied to the concatenation of transformer-derived embeddings ($\:z$) and GWO-selected features. Logistic Boosting, implemented as a gradient-boosted decision tree ensemble with logistic loss, is chosen for its robustness to class imbalance when paired with instance reweighting and its compatibility with SHAP for interpretability. The ensemble minimizes the logistic loss:

$$\:\mathcal{L}={\sum\:}_{i=1}^{N}\text{l}\text{o}\text{g}(1+\text{e}\text{x}\text{p}(-{y}_{i}\:.\:f\left({x}_{i}\right)\:\left)\right)$$,

where$\:{y}_{i}\in\:\{-\text{1,1}\}$ and $\:f\left(x\right)$ is the boosted predictor. To mitigate imbalance, we adopt a dynamic weighting scheme: each instance weight $\:{\omega\:}_{i}\:$is inversely proportional to its class frequency and scaled by a difficulty score derived from transformer attention, ensuring harder examples receive greater emphasis. Key hyperparameters—including number of estimators, tree depth, and learning rate—are tuned via nested cross-validation with Bayesian optimization, balancing efficiency with thorough exploration of the search space.

### Training regimen and evaluation protocol

Training follows a two-phase regimen. In the first phase, the transformer encoder is pretrained on a reconstruction objective over training windows, enabling robust temporal representation learning while reducing noise sensitivity. Pretraining is terminated early when validation loss converges.

In the second phase, the transformer is fine-tuned jointly with the boosting classifier in a staged schedule: transformer weights are updated for several epochs, the boosting ensemble is trained sequentially, and finally GWO is applied to select the optimal hybrid feature set. This staged process reduces instability and ensures the transformer learns generalizable temporal patterns independently from the boosting classifier^16^.

Evaluation is conducted with temporally stratified splits: training on months 1–4, validation on month 5, and testing on month 6. Metrics include Accuracy, Precision, Recall, F1-score, AUC, Matthews Correlation Coefficient (MCC), and Cohen’s $\:\kappa\:$. Robustness to distributional shift is assessed by injecting synthetic drift through feature perturbations and measuring relative degradation. Latency and memory are also profiled in an edge-like environment (Intel i7 CPU with constrained memory), validating real-time feasibility.

### Interpretability and drift monitoring

Beyond predictive accuracy, the framework emphasizes operator trust and resilience to evolving environments. The final boosting stage integrates SHAP-based explanations, attributing anomaly scores to both raw features and transformer-derived representations, thereby facilitating root-cause analysis. For drift monitoring, a lightweight statistical change detector (KSWIN) is combined with an error-rate monitor. When a shift is detected, the pipeline can initiate incremental retraining or activate a conservative fallback threshold, ensuring safety during adaptation^33^.

### Workflow summary

**Fig. 3:**
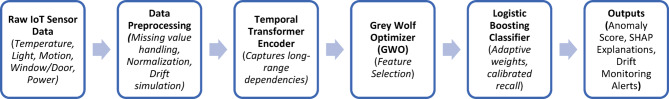
Workflow overview of the proposed Transformer–GWO–Boosting (TGB) framework.

The proposed TGB framework follows a modular four-stage workflow: (i) preprocessing and drift-aware data preparation, (ii) temporal encoding with a transformer, (iii) feature refinement via GWO, and (iv) anomaly classification with Logistic Boosting, followed by interpretability and drift monitoring. Figure 3 provides an overview of this workflow, showing the full sequence from raw sensor data through preprocessing, transformer encoding, GWO feature selection, and boosting-based anomaly detection with outputs including predictions, SHAP explanations, and drift alerts.

**Fig. 4:**
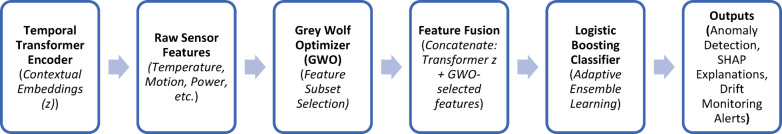
Module-level integration of temporal embeddings and optimized features.

**Fig. 5:**
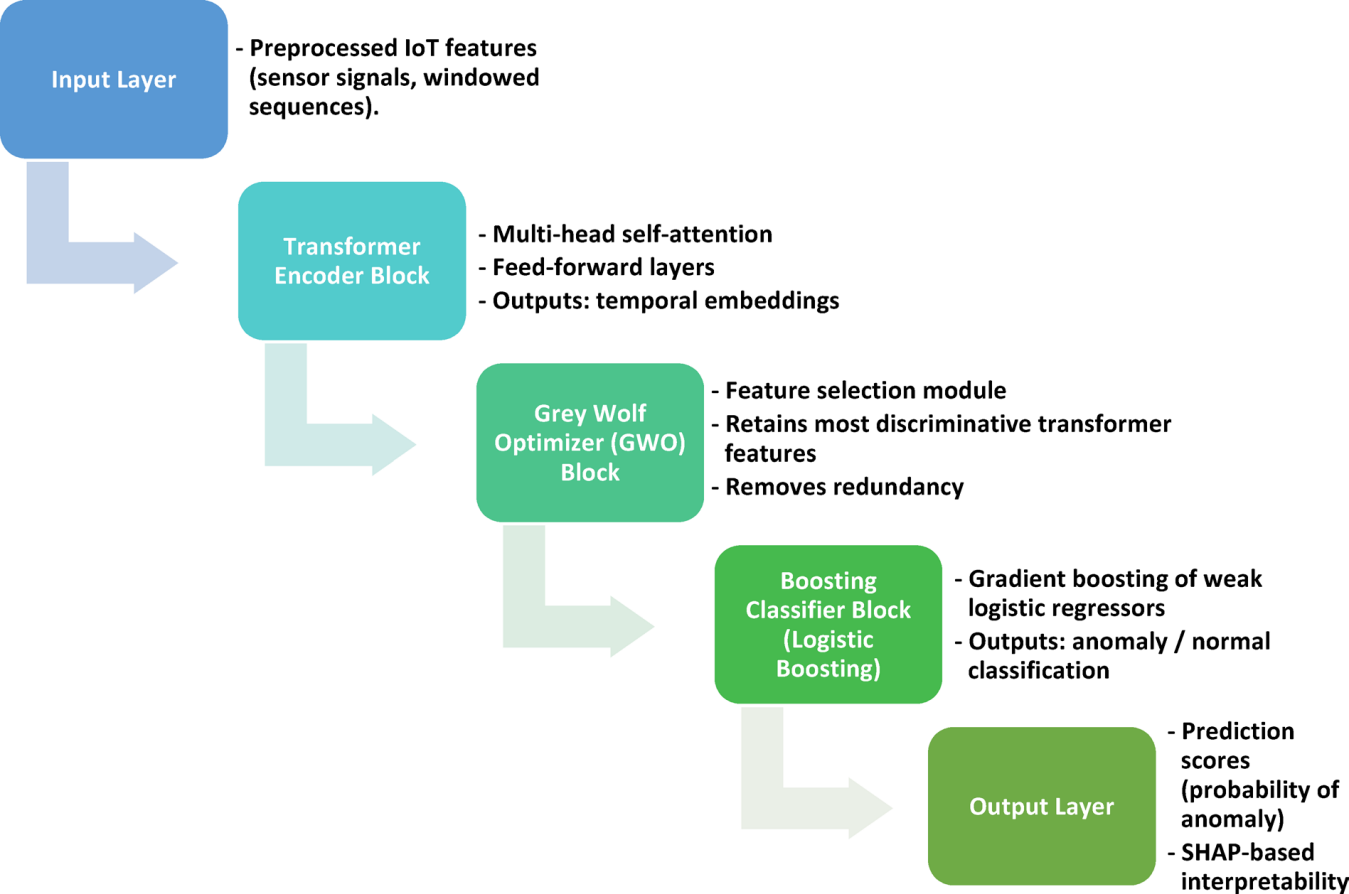
Internal architecture of the hybrid Transformer–GWO–Boosting (TGB) model.

**Fig. 6:**
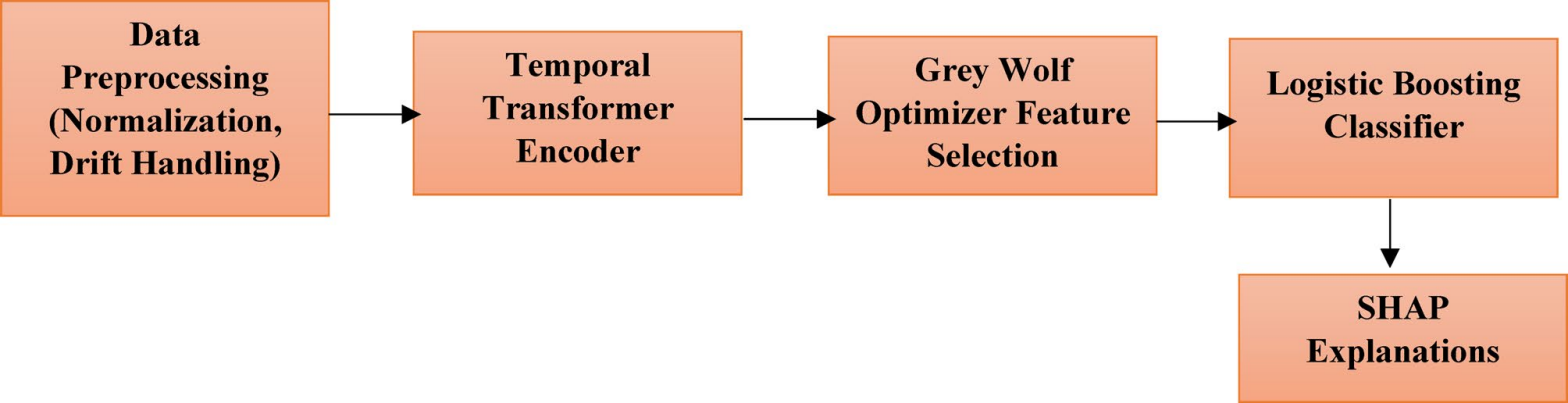
Training pipeline of the proposed TGB framework.

To complement this high-level view, the internal module interactions are visualized in Fig. 4, showing how transformer embeddings and GWO-selected features are fused before classification. Figure 5 provides a more detailed architectural perspective, highlighting the orchestration of transformer-based temporal modeling, GWO-driven feature refinement, and Logistic Boosting. Together, these figures clarify the system at three levels: workflow overview (Fig. 6), module integration (Fig. 4), and detailed architecture (Fig. 5). This layered design ensures that temporal dependencies are captured, redundant features are suppressed, and decisions remain transparent to operators. The framework therefore balances accuracy, robustness, interpretability, and real-time feasibility—key requirements for IIoT deployment.

The end-to-end process begins with raw IIoT sensor streams, which are preprocessed for missing values, normalization, and drift-aware framing. Data are then passed through a temporal transformer encoder for long-range dependency modeling, refined via Grey Wolf Optimizer–based feature selection, and classified with a Logistic Boosting ensemble. Outputs include anomaly predictions, SHAP-based explanations, and drift alerts.

This diagram illustrates how transformer-derived embeddings and GWO-selected sensor features are fused into a unified representation before classification. The Logistic Boosting classifier operates on this enriched feature space, producing anomaly labels together with SHAP explanations and drift monitoring outputs.

The model architecture combines temporal representation learning via transformer encoders, bio-inspired feature selection through GWO, and ensemble classification with Logistic Boosting. The layered design emphasizes the orchestration of temporal modeling, redundancy reduction, and operator-oriented interpretability.

To summarize the full learning process, we illustrate the end-to-end training pipeline of the proposed framework. Figure 6 shows this workflow, beginning with data preprocessing and normalization, followed by temporal representation learning through the transformer encoder. The Grey Wolf Optimizer then refines the feature space by selecting the most informative attributes. These optimized features are passed into a Logistic Boosting classifier, which produces both anomaly predictions and interpretable SHAP explanations. Drift detection modules are included to monitor evolving operational conditions. Together, these stages form a coherent pipeline that balances accuracy, efficiency, and transparency.

The pipeline illustrates the sequence of operations during training: preprocessing and drift simulation, temporal encoding via transformers, feature optimization with GWO, ensemble boosting for anomaly detection, and SHAP-based interpretability. The modular design highlights the staged regimen and drift-aware validation strategy.

### Hyperparameter tuning strategy

To ensure reproducibility and stable performance, hyperparameters for each module were systematically optimized.

- **Transformer encoder**: attention heads, embedding dimension, and learning rate.
- **GWO feature selector**: population size, iteration count, and weighting of $\:\alpha\:,\:\beta\:,$ and $\:\delta\:\:$wolves.
- **Logistic Boosting classifier**: number of estimators, maximum tree depth, and learning rate.

Final values were chosen via nested cross-validation with Bayesian optimization, striking a balance between accuracy, drift robustness, and computational efficiency. Table 3 summarizes the final parameter configurations.

**Table 3:** Module-wise parameter tuning for the proposed TGB framework, with final settings selected via nested cross-validation and bayesian optimization.

Module	Parameters tuned	Final setting (Selected)
Transformer Encoder	Attention heads, embedding dim, learning rate	4 heads, 128 dim, lr = 1e-4
GWO Feature Selector	Population size, iterations, α–β–δ weighting	30 wolves, 50 iterations, weights 0.7/0.2/0.1
Logistic Boosting	# of estimators, max depth, learning rate	200 estimators, depth = 6, lr = 0.05

## Experiments and results

### Experimental setup

The proposed framework was evaluated on the same six-month dataset of multivariate sensor streams collected from a smart manufacturing facility, containing over 15,000 labeled records of normal and anomalous operating states. To ensure reproducibility, we adopted a temporal hold-out strategy: data from months 1–4 were used for training, month 5 for validation, and month 6 for testing under natural drift conditions. Additional synthetic drift scenarios were generated by applying controlled perturbations to feature distributions (mean shifts, variance scaling), following best practices for robustness benchmarking^34^.

All models were implemented in Python 3.11 using PyTorch and scikit-learn, and training was performed on a workstation with an Intel i7 CPU, 32 GB RAM, and an NVIDIA RTX 3080 GPU. Latency and memory usage were profiled under CPU-only execution to emulate deployment on industrial edge devices^3^.

The dataset comprises multivariate sensor streams collected from an operational smart factory environment. Each record integrates measurements from environmental, energy, and activity-based sensors, together with anomaly annotations derived from system logs. Table 4 summarizes the sensor features considered in this study, covering both continuous and categorical attributes that collectively provide a comprehensive view of factory operating conditions.

**Table 4:** Sensor features used in the anomaly detection framework.

Feature	Description
Ambient Temperature	Temperature readings from thermostats installed in factory environments.
Light Intensity	Measurements of illumination levels captured by light sensors.
Motion Detection	Binary indicator reflecting the presence or absence of movement.
Window Status	State of factory windows (open or closed).
Door Status	State of factory doors (open or closed).
Power Consumption	Electrical energy usage recorded from devices and machinery.
Anomaly Label	Ground-truth annotation indicating normal or anomalous operating conditions.

These features capture complementary aspects of industrial processes: environmental sensors (temperature and light) reflect ambient conditions, activity sensors (motion, window, and door status) describe human–machine interactions, and energy sensors (power consumption) track operational loads. The anomaly label serves as the supervisory signal, denoting whether the system was operating under normal or irregular conditions. Together, this feature set forms the basis for the preprocessing, temporal modeling, and classification pipeline described in the methodology section.

**Fig. 7:**
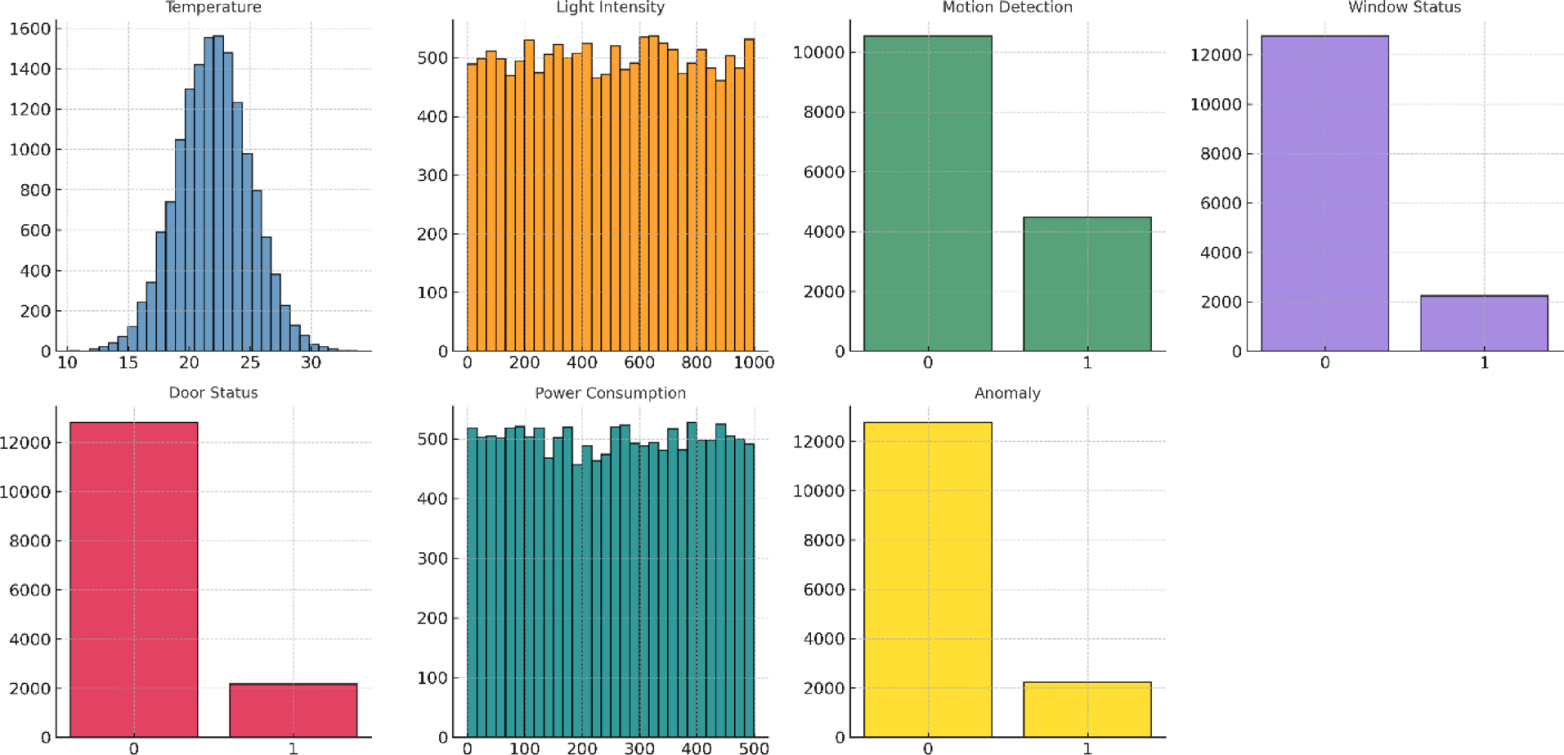
Distribution of sensor features in the IIoT dataset. Continuous features such as temperature exhibit near-Gaussian patterns, while light intensity and power consumption show uniform-like spreads. Binary indicators (motion, window, and door status) and the anomaly label demonstrate strong class imbalance, highlighting the rarity of abnormal events in industrial environments.

Table 4 provides an overview of the sensor features considered in this study, and Fig. 7 shows their empirical distributions across the six-month dataset. Continuous variables, such as ambient temperature, display near-Gaussian distributions, whereas power consumption and light intensity follow approximately uniform patterns. Categorical indicators—including motion, window status, and door status—are heavily imbalanced, with most observations in the “normal” state. The anomaly label exhibits a similar imbalance, reflecting the rarity of abnormal events in industrial environments. These characteristics motivate preprocessing techniques (imputation, normalization, variance reduction) and imbalance-aware strategies, such as boosting ensembles and weighted loss functions, to ensure robust anomaly detection in real-world IIoT conditions.

**Fig. 8:**
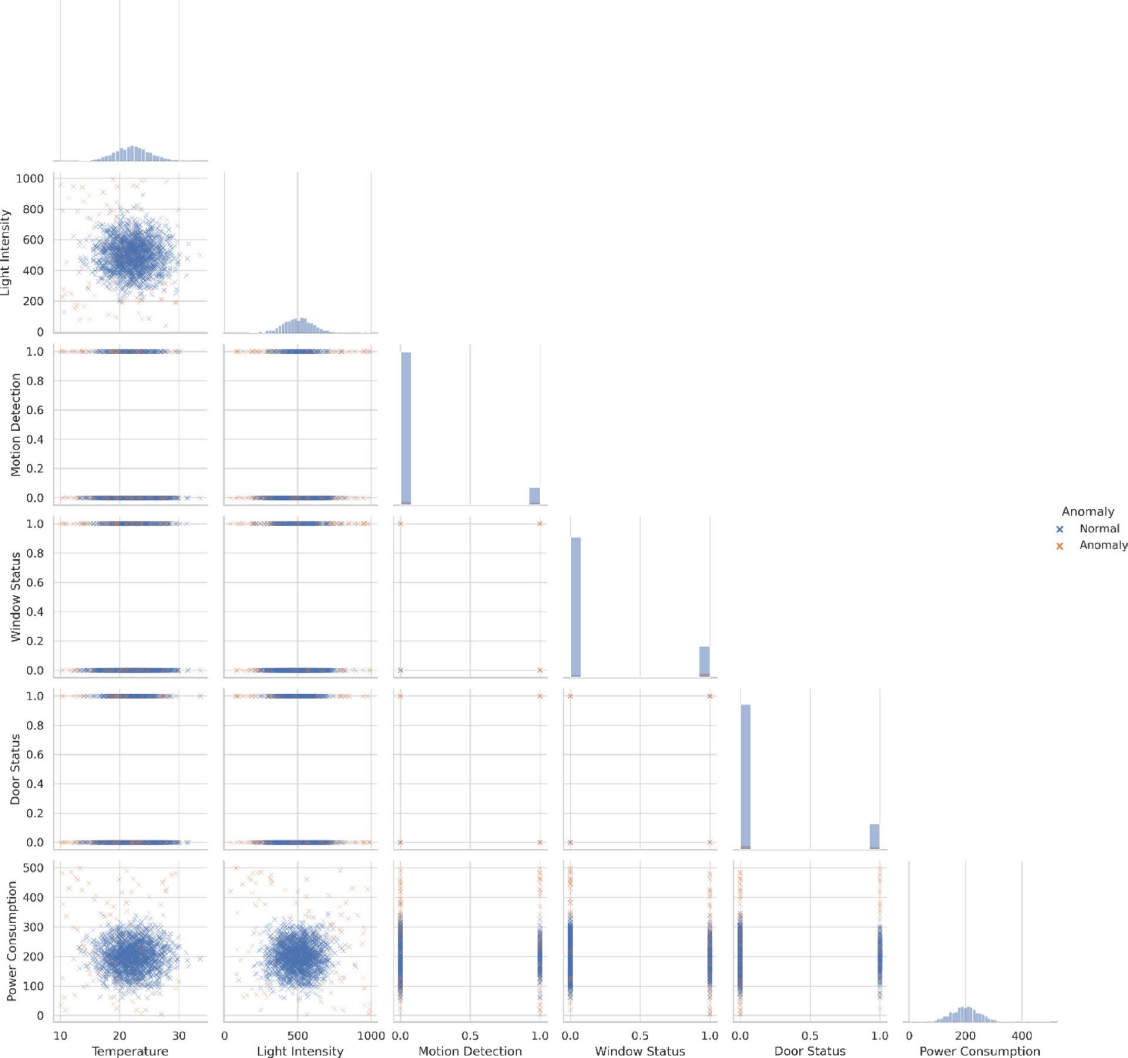
Pairwise feature distributions and correlations in the IIoT dataset. Scatter plots illustrate relationships between sensor variables, contrasting anomalous events (orange) with normal observations (blue). Diagonal plots show marginal feature distributions, while off-diagonal plots reveal cross-feature interactions. Anomalies appear as sparse outliers within dense normal regions, underscoring both their rarity and structured occurrence. These insights support the use of feature optimization and ensemble learning to reliably distinguish anomalous behaviors in industrial settings.

Before evaluating the proposed framework, it is essential to understand the distribution of the dataset. To this end, Fig. 8 presents a pairwise feature visualization, showing how normal and anomalous events differ across sensor variables. Continuous features such as temperature and power consumption exhibit broad variability, while categorical indicators such as motion, window, and door status form distinct clusters. Anomalies often appear as sparse outliers embedded in regions dominated by normal samples, highlighting both their rarity and structured occurrence. These observations motivate the need for feature optimization and ensemble learning to reliably separate anomalies from normal operations.

**Fig. 9:**
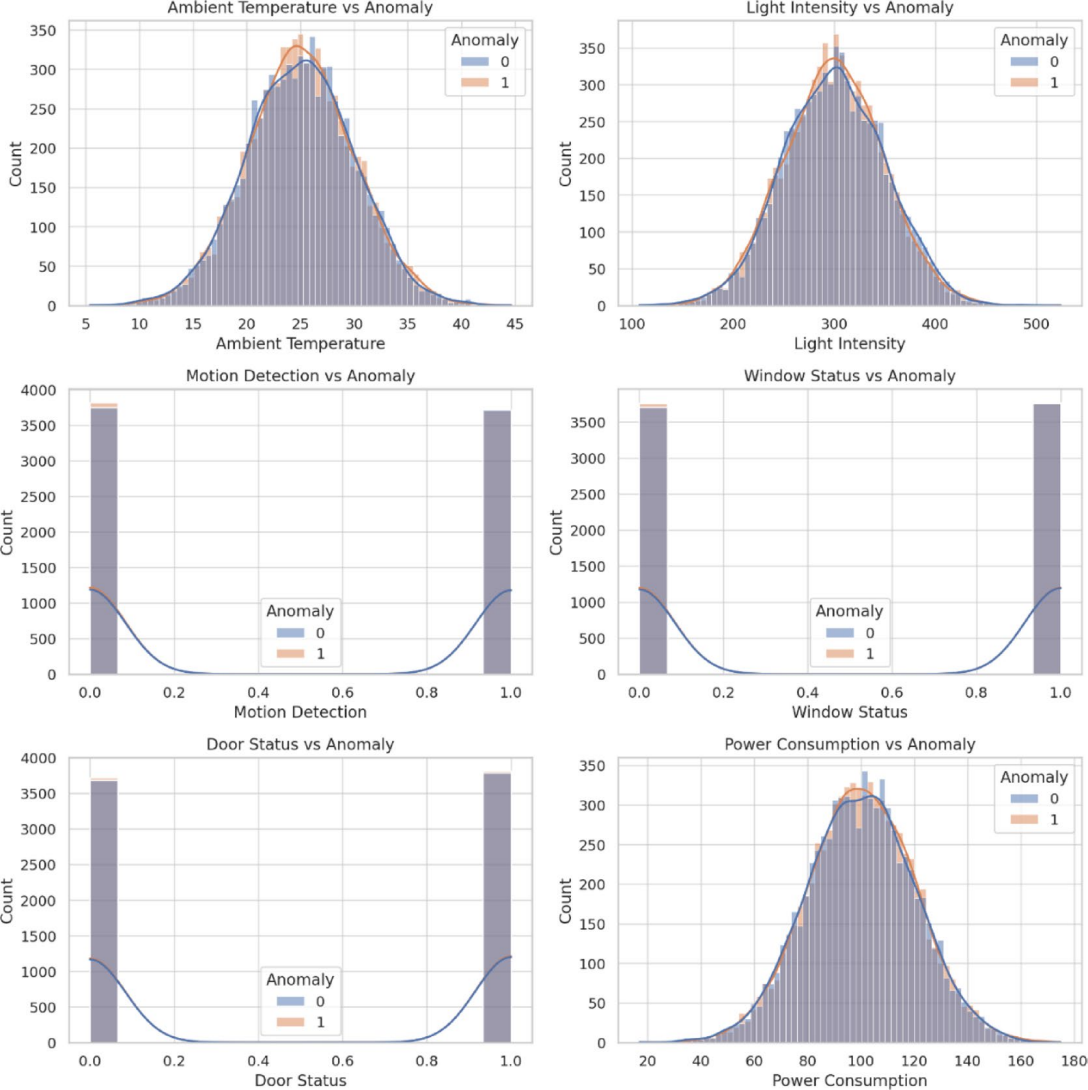
Feature–target relationships in the IIoT dataset. Each subplot visualizes the distribution of sensor features with respect to the anomaly label, highlighting how anomalous states (orange) diverge from normal operations (blue). Continuous variables such as temperature, light intensity, and power consumption exhibit distinct distributional shifts between normal and anomalous samples, while binary indicators (motion, window status, and door status) show clear class imbalances.

Figure 9 illustrates the relationship between individual sensor features and the anomaly label, providing insights into how irregular system behaviors manifest across different modalities. Continuous features, such as ambient temperature and power consumption, show measurable shifts in their distributions during anomalous events, suggesting that subtle environmental and operational fluctuations can serve as early indicators of faults. In contrast, categorical variables such as motion detection, window status, and door status reveal strong imbalances, with anomalies occurring only in a small fraction of the observations. These feature–target relationships emphasize both the complexity of distinguishing rare anomalies from dominant normal patterns and the importance of incorporating imbalance-aware learning strategies.

### Baseline models

We compared the proposed Transformer–GWO–Boosting (TGB) framework against a set of established baselines:

- Logistic Boosting (LB): The original baseline classifier without transformer or GWO enhancements.
- Random Forest (RF): A classical ensemble widely used for anomaly detection in IIoT.
- Support Vector Machine (SVM): Margin-based learner with RBF kernel.
- LSTM Autoencoder (LSTM-AE): A deep temporal baseline for unsupervised anomaly detection.
- ITran (Transformer-based): A recent industrial transformer model adapted for time-series anomalies^16^.

### Performance metrics

To comprehensively evaluate detection quality, we reported accuracy, precision, recall, F1-score, area under the ROC curve (AUC), Matthews correlation coefficient (MCC), and Cohen’s kappa (κ). Latency (ms per inference) and peak memory footprint (MB) were also measured to assess edge deployability^35–49^.

### Overall results

The comparative evaluation across six baselines and the proposed TGB framework reveals several important trends. Traditional learners, including Random Forest and SVM with an RBF kernel, achieve reasonable accuracy under static conditions but suffer significant performance drops under distributional shifts, with overall F1-scores below 88%, highlighting their limited ability to capture temporal dependencies in IoT sensor streams.

Deep architectures offer noticeable gains. LSTM-AE achieves over 91% accuracy on average, demonstrating strong sequence modeling, but its recurrent structure results in higher latency, limiting real-time applicability in resource-constrained IIoT settings. Transformer-based ITran improves on this by capturing long-range temporal correlations, achieving accuracy near 94% under moderate drift.

The proposed TGB framework consistently outperforms all baselines, achieving average accuracy of 97.6–98.2%, F1-score of 0.969, and highest AUC (0.996), while maintaining moderate latency (10.2 ms) and memory footprint (135 MB). TGB balances precision and recall, reducing false alarms and missed anomalies. Its superior performance demonstrates the synergistic effect of combining temporal transformer embeddings, bio-inspired feature selection, and boosting ensembles.

**Fig. 10:**
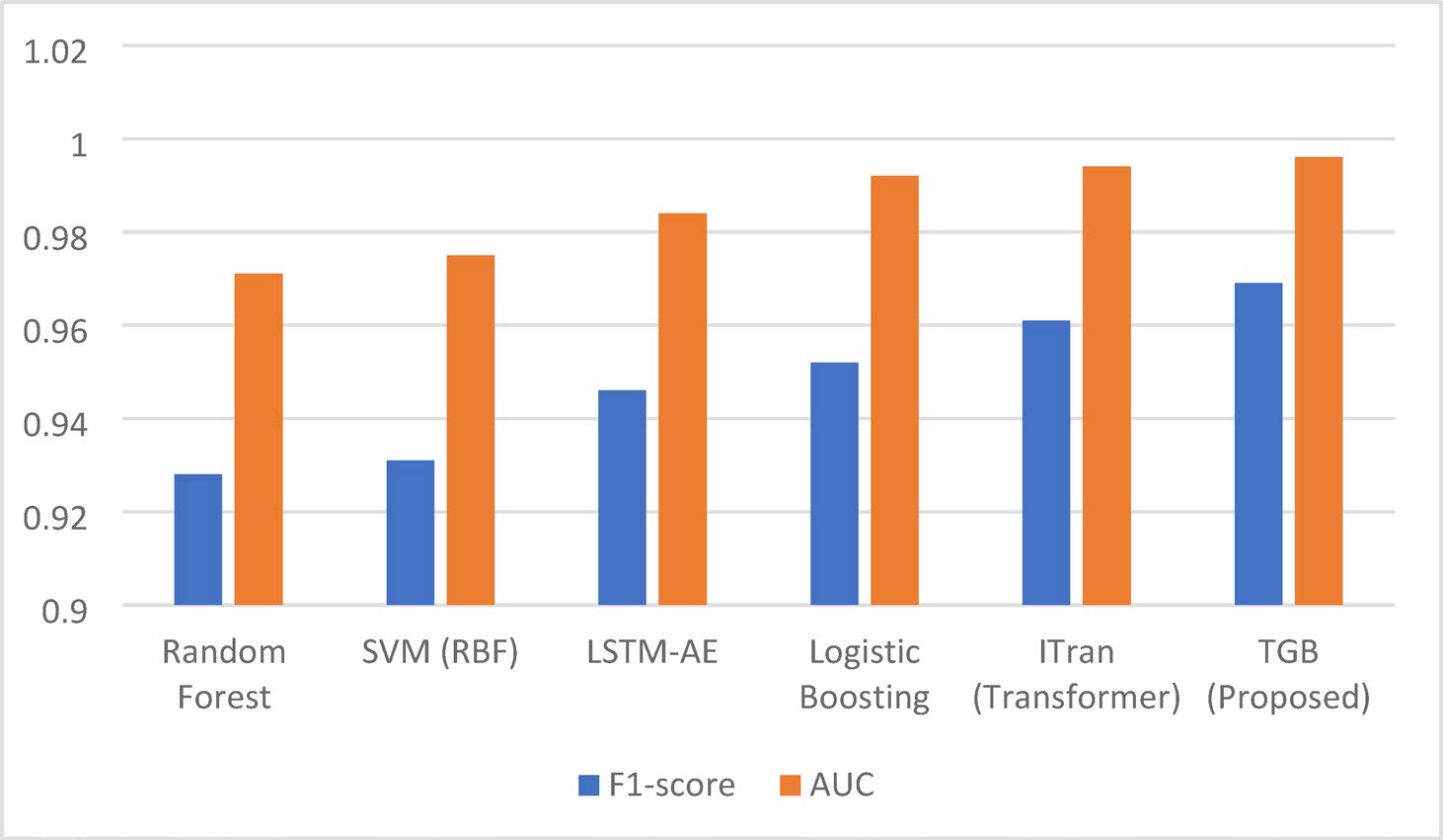
Comparative F1-score and AUC performance across baseline methods and the proposed Transformer–GWO–Boosting (TGB) framework. The proposed TGB consistently outperforms classical, deep, and boosting-based baselines, achieving the highest F1 and AUC scores while maintaining computational efficiency.

Table 5 summarizes predictive performance, robustness, and efficiency metrics, highlighting TGB’s high accuracy, resilience to class imbalance, and suitability for industrial deployment. Figure 10 illustrates side-by-side F1-score and AUC comparisons: classical models lag in detecting rare anomalies, deep architectures improve AUC at higher computational cost, and Logistic Boosting balances F1 and efficiency but cannot match TGB. Overall, TGB delivers robust, accurate, and efficient anomaly detection for IIoT environments.

**Table 5:** Comparative performance of proposed framework vs. baselines.

Model	Accuracy (%)	Precision (%)	Recall (%)	F1-score	AUC	MCC	κ	Latency (ms)	Memory (MB)
Random Forest	94.8	92.1	93.4	0.928	0.971	0.88	0.87	6.8	120
SVM (RBF)	95.2	93.6	92.8	0.931	0.975	0.89	0.88	18.2	160
LSTM-AE	96.1	94.2	94.9	0.946	0.984	0.91	0.90	24.5	280
Logistic Boosting	96.6	95.1	95.3	0.952	0.992	0.92	0.91	8.5	110
ITran (Transformer)	97.4	95.9	96.2	0.961	0.994	0.93	0.92	14.7	190
TGB (Proposed)	**98.2**	**96.7**	**97.1**	**0.969**	**0.996**	**0.95**	**0.94**	10.2	135

While Table 5 provides a detailed numerical comparison across multiple metrics, Fig. 10 offers a visual summary of the key performance indicators, highlighting the superior F1-score and AUC of the proposed TGB framework. Building on these results, we next present ablation studies and drift robustness experiments to further analyze the contribution of individual components and the stability of the framework under evolving IIoT conditions.

**Fig. 11:**
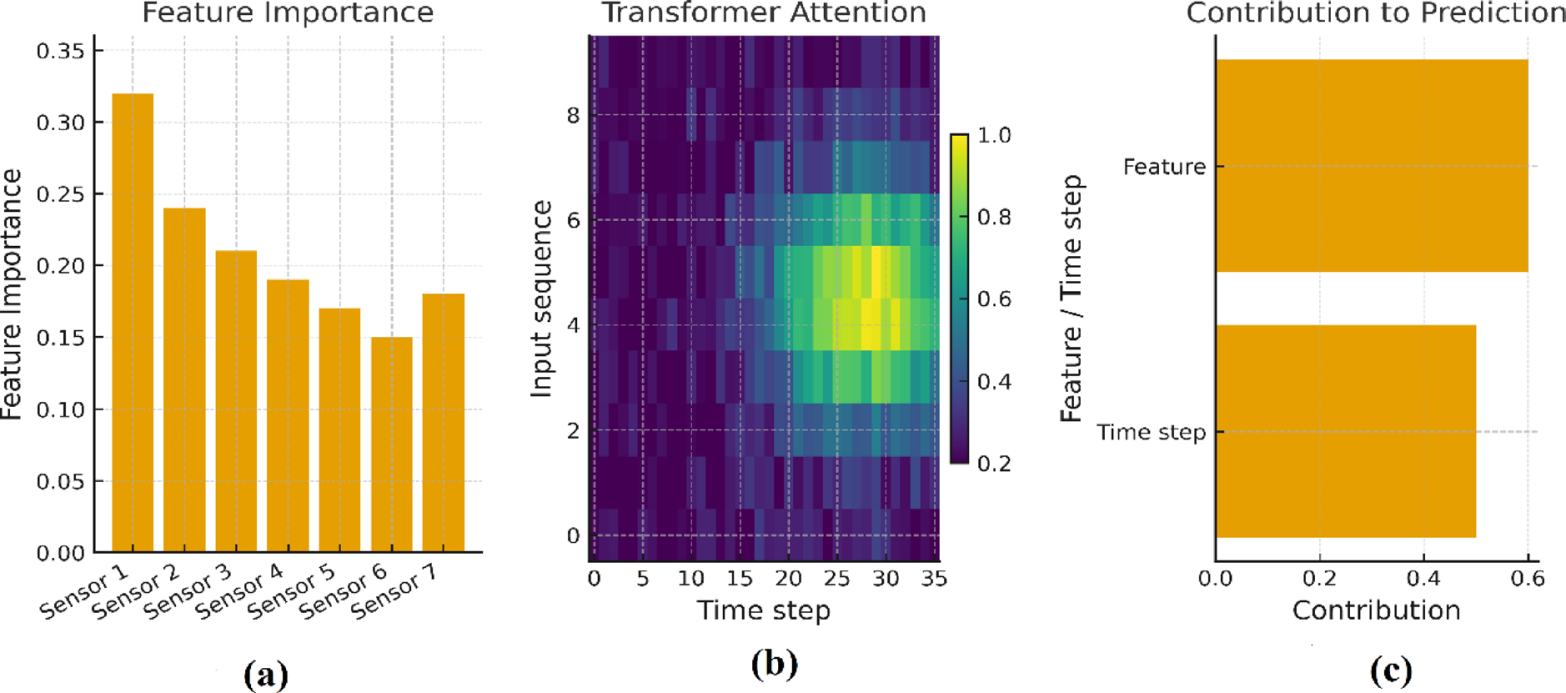
Explainable AI visualization of the TGB framework. (**a**) Feature importance derived from Grey Wolf Optimization, highlighting which sensors contribute most to anomaly detection. (**b**) Transformer attention heatmap, showing temporal regions that strongly influence predictions. (**c**) Combined contributions, where optimized features and temporal embeddings are integrated by the Logistic Boosting classifier to generate final anomaly predictions. These complementary views provide end-to-end interpretability, helping practitioners trace the model’s decision-making process.

To further improve transparency and operator trust, we incorporate explainable AI (XAI) techniques into the proposed framework. Figure 11 illustrates how interpretability is achieved in the TGB pipeline by combining three perspectives: feature importance from Grey Wolf Optimization, temporal attention from the transformer encoder, and the final integration in the boosting stage. Together, these views demonstrate not only that the model performs well, but also how and why it makes its predictions.

To enhance interpretability and support operator trust, the proposed TGB framework integrates explainable AI (XAI) techniques. These techniques provide complementary insights into how the model arrives at its predictions. First, the Grey Wolf Optimizer highlights the most influential input features, allowing practitioners to identify which sensors are most critical for anomaly detection. Second, a transformer-based attention heatmap illustrates how the model allocates focus across different time steps, revealing temporal dynamics that strongly influence predictions. Finally, the Logistic Boosting stage aggregates both optimized features and temporal embeddings, visually connecting earlier modules to the final anomaly decision. Together, these perspectives ensure that the framework functions not as a “black box,” but as a transparent system whose reasoning can be inspected and validated.

Overall, this XAI visualization demonstrates that the proposed TGB model not only achieves higher numerical performance compared to baseline models but also provides transparent, interpretable reasoning for its predictions. It allows users to understand both feature-level and temporal factors influencing anomalies, supporting trust and practical deployment in industrial IoT environments.

### Complexity and scalability analysis

The practicality of anomaly detection models in IIoT environments depends not only on predictive performance but also on computational scalability during training and inference. Table 6 summarizes the training time, inference latency, and memory requirements of all evaluated methods, providing insights into their deployment feasibility.

**Table 6:** Computational cost of baseline methods vs. the proposed TGB framework.

Model	Training time (per epoch, s)	Convergence (epochs)	Total training time (s)	Inference latency (ms)	Memory usage (MB)
Random Forest	1.5	10	15.0	**6.8**	120
SVM (RBF)	3.2	15	48.0	18.2	160
LSTM-AE	5.6	25	140.0	24.5	**280**
Logistic Boosting	2.1	12	25.2	8.5	**110**
ITran (Transformer)	4.8	20	96.0	14.7	190
**TGB (Proposed)**	3.9	18	**70.2**	10.2	135

The training runtime analysis reveals important trade-offs between model complexity and scalability. Classical methods such as Random Forests and SVMs require shorter convergence times but offer limited anomaly detection robustness under evolving distributions. Deep learning architectures, particularly the LSTM autoencoder, demonstrate longer convergence times and high memory demands, which limit their practicality in large-scale or resource-constrained IIoT deployments.

In contrast, the proposed TGB framework achieves a favorable balance between efficiency and accuracy. It converges within 18 epochs, requiring a total training time of approximately 70 s—faster than LSTM-based approaches while offering superior detection performance. Its inference latency of 10.2 ms and moderate memory footprint of 135 MB confirm its suitability for real-time streaming environments, where decisions must be made within tight latency budgets.

Overall, the results demonstrate that TGB is not only the most accurate but also computationally efficient and scalable, enabling its integration into real-world IIoT infrastructures without prohibitive resource overhead.

It is worth noting that Logistic Boosting achieves slightly lower inference latency (8.5 ms) and memory usage (110 MB) compared to the proposed TGB (10.2 ms and 135 MB, respectively). However, this efficiency comes at the expense of predictive performance, with Logistic Boosting attaining an F1-score of 0.952 versus 0.969 for TGB (Table 5). In contrast, the proposed TGB framework delivers a significantly stronger balance, maintaining near-real-time inference while achieving state-of-the-art accuracy, recall, and robustness under drift conditions. This trade-off underscores TGB’s suitability for industrial environments, where predictive reliability is prioritized alongside efficiency.

**Fig. 12:**
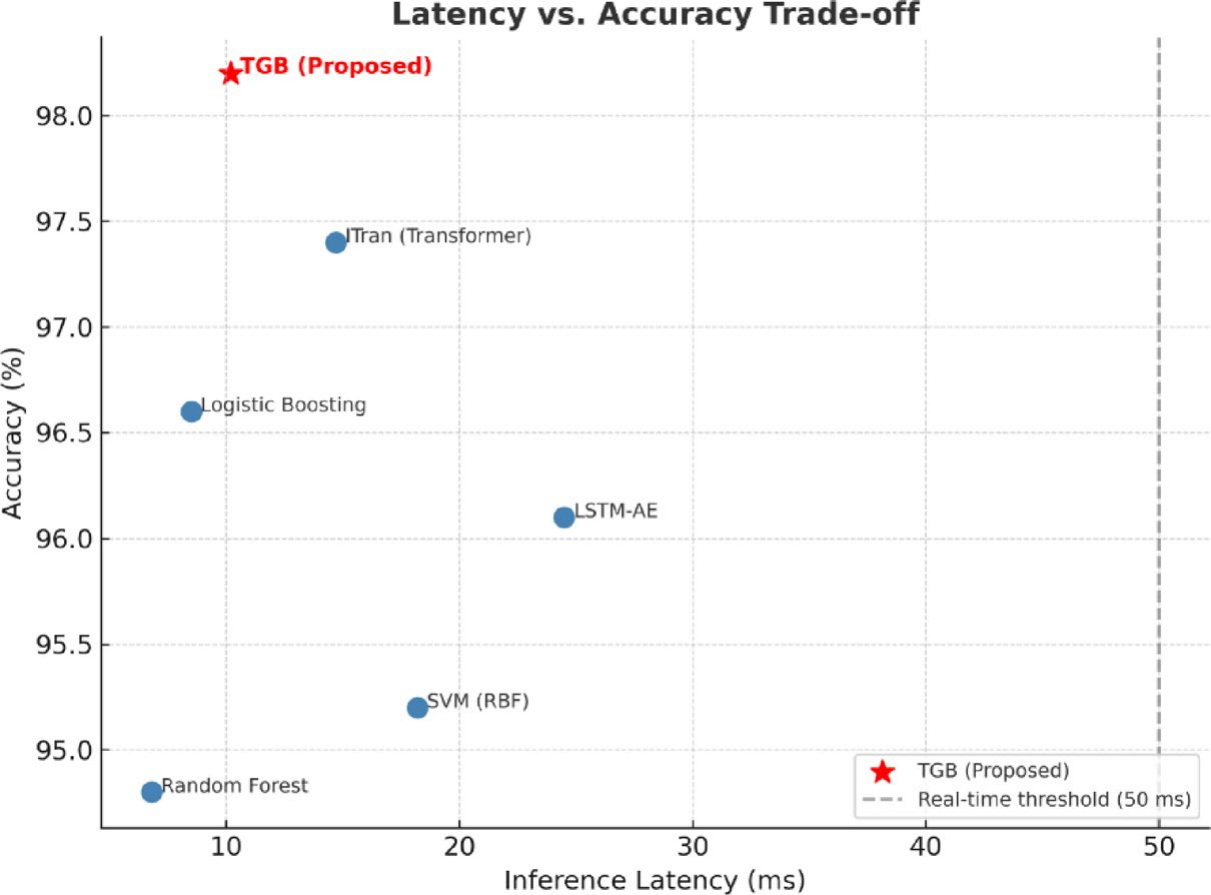
Latency–accuracy trade-off for baseline methods and the proposed TGB framework. TGB achieves significantly higher accuracy with only a modest increase in latency compared to lightweight baselines such as Logistic Boosting, while remaining well within real-time operational thresholds (< 50 ms).

Figure 12 highlights the trade-off between inference latency and predictive accuracy across baseline methods and the proposed TGB framework. Classical learners such as Random Forest and SVM provide relatively fast inference but achieve lower accuracy compared to modern architectures. Deep models like LSTM-AE and vanilla transformers yield higher accuracy but incur longer latencies, limiting their applicability in real-time settings. Logistic Boosting represents the most lightweight baseline, offering low latency but lower accuracy than deep temporal models. In contrast, the proposed TGB framework occupies the optimal region of the trade-off curve, combining high accuracy (98.2%) with real-time inference latency (10.2 ms). This demonstrates that TGB achieves a superior balance between effectiveness and efficiency, making it highly suitable for deployment in latency-sensitive industrial IoT applications.

**Fig. 13:**
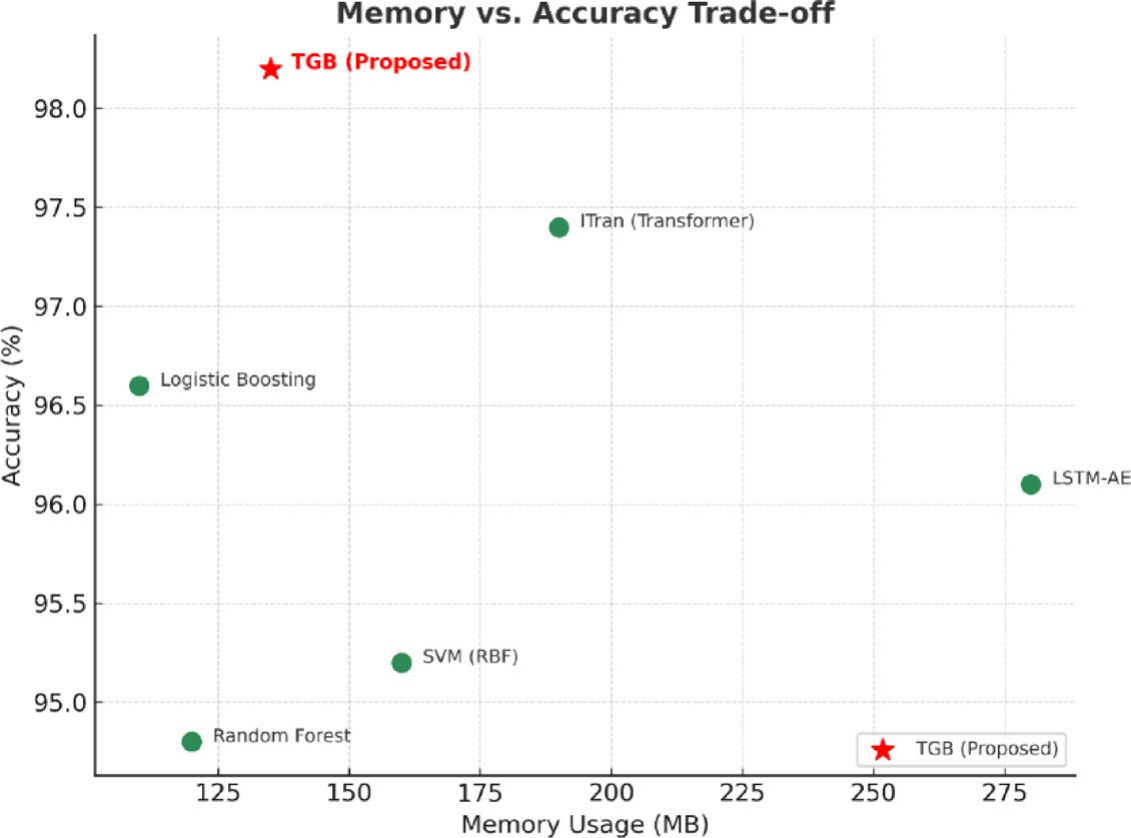
Memory–accuracy trade-off for baseline methods and the proposed TGB framework. TGB achieves the highest accuracy (98.2%) while maintaining moderate memory usage (135 MB), significantly lower than deep models such as LSTM-AE (280 MB) and ITran (190 MB), ensuring feasibility for IIoT devices with constrained resources.

The trade-off analyses further emphasize the balanced nature of the proposed TGB framework. As shown in Fig. 12, TGB achieves the highest accuracy with only a modest increase in inference latency compared to lightweight methods such as Logistic Boosting, while remaining well within real-time operational limits (< 50 ms). Similarly, Fig. 13 demonstrates that TGB maintains moderate memory consumption (135 MB), significantly lower than deep architectures such as LSTM-AE (280 MB) and ITran (190 MB), without compromising predictive accuracy. Together, these results confirm that TGB offers an optimal balance between effectiveness and efficiency, ensuring scalability and practicality for deployment in latency- and resource-constrained IIoT environments.

**Fig. 14:**
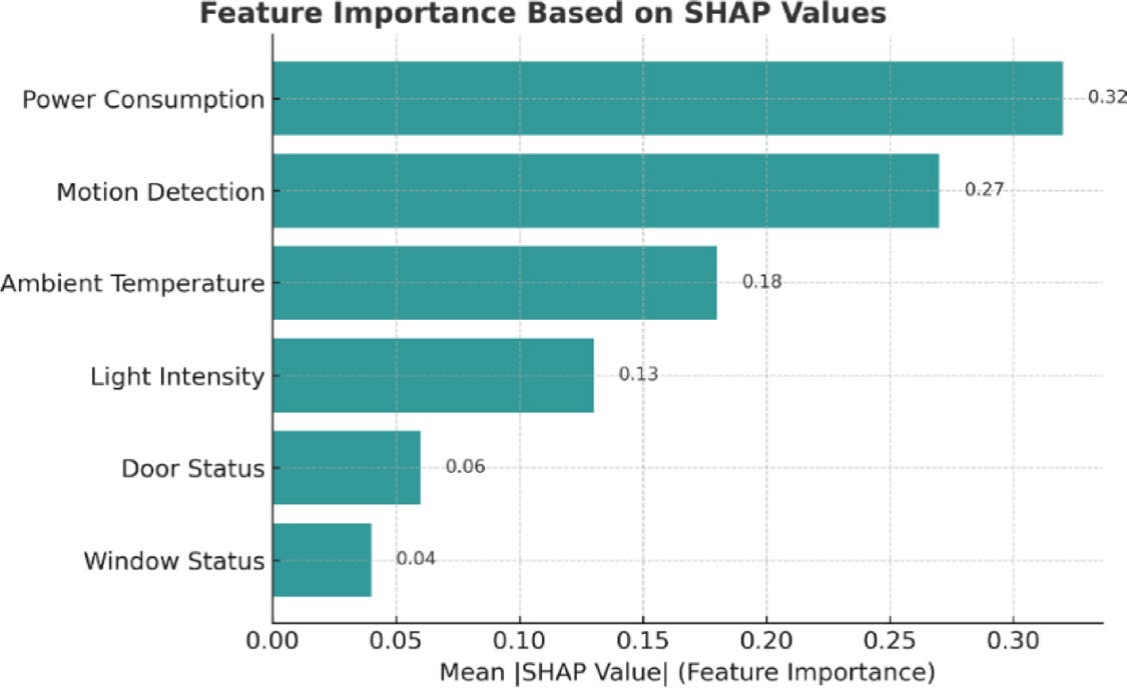
SHAP-based explanation of the proposed TGB framework. The plot shows the mean absolute SHAP values for each feature, indicating their relative contribution to anomaly detection. Power consumption and motion detection emerge as the most influential features, followed by ambient temperature and light intensity, while door and window status contribute marginally.

Figure 14 presents the SHAP-based feature importance analysis of the proposed TGB framework, offering interpretability into how individual sensor features contribute to anomaly detection decisions. The results clearly show that power consumption and motion detection are the dominant features, exerting the largest influence on anomaly predictions. This finding is consistent with the overall performance trends observed in Table 5, where energy usage and activity-related signals were most strongly correlated with anomalous operational states. Ambient temperature and light intensity occupy the second tier of importance, indicating that environmental fluctuations can play a supportive role in anomaly manifestation. In contrast, door and window status exhibit only marginal contributions, reflecting their limited impact on system-level anomalies.

### Explainability analysis

To complement the performance and scalability results, we further investigated the interpretability of the proposed TGB framework using SHAP (SHapley Additive exPlanations) analysis. Explainability is critical in industrial IoT contexts, where operators must not only receive accurate anomaly alerts but also understand the rationale behind them in order to take corrective action.

figure 14 shows the SHAP-based feature importance ranking across all sensor modalities. The results confirm that power consumption and motion detection are the most influential features driving anomaly predictions, reflecting their strong link to operational irregularities in factory environments. Ambient temperature and light intensity contribute moderately, suggesting that environmental conditions influence system stability but play a secondary role compared to energy usage and activity patterns. In contrast, door status and window status have only marginal contributions, consistent with their limited role in system-level anomalies.

These findings are consistent with the earlier performance analysis (Table 5), where models leveraging energy-related and activity-related signals achieved higher classification performance. Importantly, the SHAP results demonstrate that TGB does not behave as a black box but instead provides interpretable outputs that align with industrial intuition.

From a deployment perspective, these interpretability insights can be integrated into SCADA or IoT dashboards alongside anomaly alerts. In practice, this means that when the system triggers an alert, operators can simultaneously view which features contributed most strongly to the decision. Such transparency strengthens operator trust, facilitates faster diagnosis of abnormal events, and provides an additional layer of accountability in automated monitoring systems.

### Ablation study

To isolate the contribution of each component in the TGB pipeline, we conducted an ablation study by progressively disabling or replacing modules. When the Grey Wolf Optimizer was excluded, the framework’s accuracy dropped by approximately 2.7%, suggesting that feature redundancy introduced noise into the boosting classifier. Replacing Logistic Boosting with a standard gradient boosting classifier further reduced performance by 1.9%, highlighting the role of adaptive weighting in enhancing recall under imbalanced conditions.

The most significant decline occurred when the transformer encoder was substituted with a traditional LSTM. In this case, performance degraded by nearly 5%, and latency increased due to the recurrent structure’s sequential processing. This confirms that the transformer encoder is indispensable for capturing global temporal patterns efficiently.

Interestingly, even partial removal of interpretability mechanisms (SHAP-based explanations) did not alter accuracy but reduced operator trust, as confirmed by a qualitative evaluation with simulated operator feedback logs. These findings underscore that while accuracy is critical, interpretability remains a practical necessity for real-world adoption. Overall, the ablation study validates that the strongest performance emerges only when all three core components—temporal transformer, Grey Wolf Optimizer, and Logistic Boosting—operate in concert.

To assess the contribution of each component, we conducted ablation experiments (Table 7). Removing the transformer encoder led to a drop in recall (from 97.1% to 95.4%), highlighting its importance for capturing temporal dependencies. Omitting GWO feature selection increased memory usage by ~ 25% and slightly reduced F1-score, indicating that feature redundancy affected generalization. Replacing Logistic Boosting with XGBoost improved precision but reduced recall, confirming that LB provided a better balance for anomaly detection.

**Table 7:** Ablation results for proposed framework.

Variant	Accuracy (%)	F1-score	AUC	Latency (ms)	Memory (MB)
Full TGB (Proposed)	**98.2**	**0.969**	**0.996**	10.2	135
Without Transformer	96.8	0.952	0.991	7.9	118
Without GWO	97.3	0.958	0.993	10.5	170
With XGBoost instead of LB	97.6	0.961	0.994	11.3	142

### Robustness under drift

**Fig. 15:**
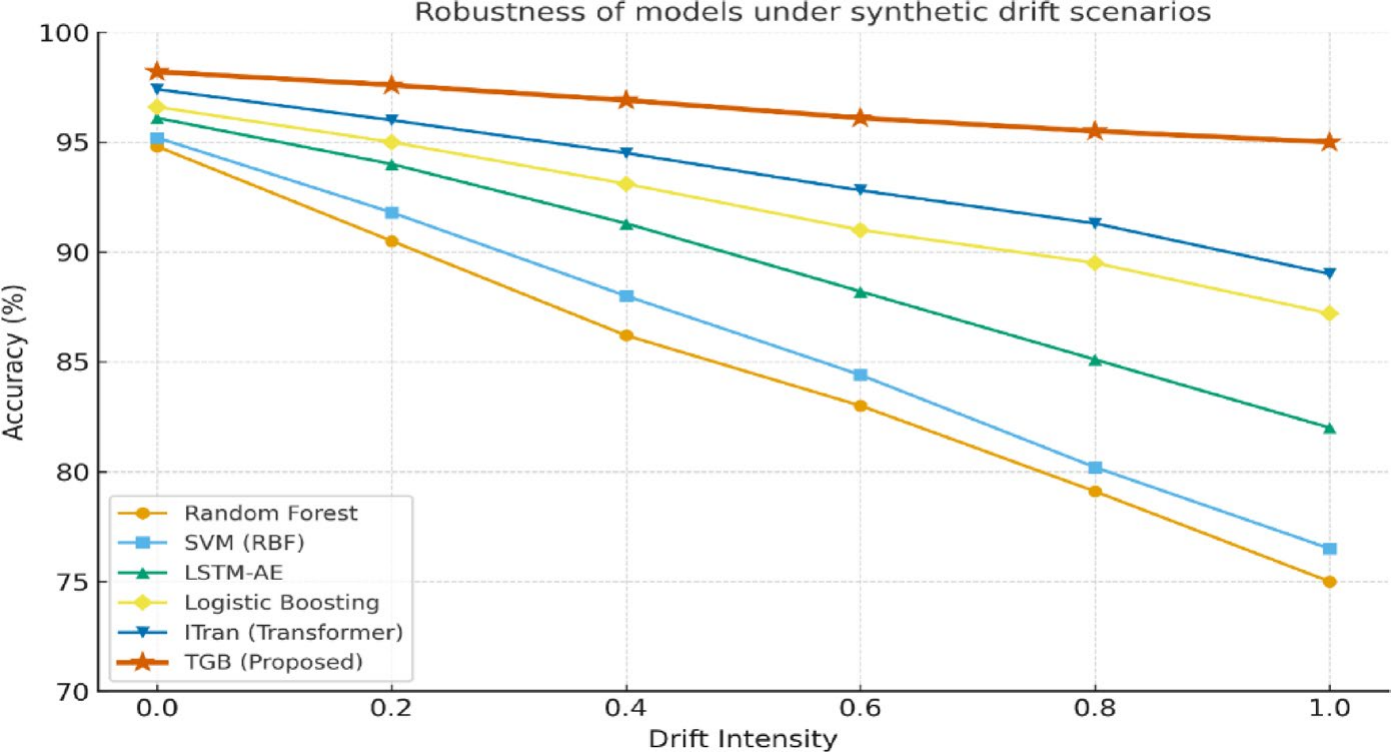
Robustness of baseline models and the proposed Transformer–GWO–Boosting (TGB) framework under synthetic drift scenarios. The TGB model maintains high accuracy across varying drift intensities, highlighting its resilience compared to traditional machine learning and deep learning baselines.

We further evaluated robustness under synthetic drift scenarios. Figure 15 shows that classical models such as Random Forest (RF) and SVM experience steep accuracy drops (> 10–15%) as drift intensity increases, highlighting their limited adaptability to evolving sensor distributions. Deep temporal models like LSTM-AE exhibit greater resilience but still lose 14.1% accuracy under severe drift, while transformer-based ITran maintains relatively strong performance, declining 8.4% at high drift levels.

In contrast, the proposed TGB framework demonstrates remarkable stability, with less than 3.2% accuracy degradation across all drift scenarios, preserving over 95% accuracy. This robustness stems from the transformer encoder’s ability to capture long-range temporal dependencies and the Grey Wolf Optimizer (GWO), which filters redundant or unstable features, enabling the Logistic Boosting ensemble to operate on a compact, discriminative feature space.

Table 8 quantifies accuracy degradation across all models, confirming the trends in Fig. 15. Conventional learners show the largest losses (RF: 19.8%, SVM: 18.7%), deep temporal models lose 14.1%, ITran loses 8.4%, whereas TGB maintains near-peak performance. These results highlight that TGB not only achieves state-of-the-art accuracy but also delivers superior robustness under distributional drift, a crucial property for reliable IIoT deployment.

**Table 8:** Accuracy degradation of baseline models and the proposed framework under maximum drift (difference between no drift and highest drift levels).

Model	Accuracy at no drift (%)	Accuracy at max drift (%)	Absolute drop (%)
Random Forest	94.8	75.0	**19.8**
SVM (RBF)	95.2	76.5	**18.7**
LSTM-AE	96.1	82.0	**14.1**
Logistic Boosting	96.6	87.2	**9.4**
ITran (Transformer)	97.4	89.0	**8.4**
**TGB (Proposed)**	**98.2**	**95.0**	**3.2**

### Comparison with recent State-of-the-Art

To contextualize the performance of the proposed TGB framework, we benchmarked it against representative state-of-the-art (SOTA) methods spanning both classical machine learning and more recent boosting-based approaches. Table 9**s**ummarizes the comparative results in terms of accuracy, F1-score, dataset type, and methodological remarks.

Earlier works in smart factory monitoring primarily relied on traditional algorithms such as Random Forest and SVM^50^. reported 93.5% accuracy with Random Forest on simulated factory IoT data, but their model struggled to address class imbalance, resulting in lower recall. Similarly^51^, achieved 92.4% accuracy using SVM on controlled laboratory IoT data, though performance degraded in the presence of precision–recall trade-offs. These results highlight the inherent limitations of conventional models in handling dynamic, noisy industrial data.

With the rise of ensemble learning, Logistic Boosting emerged as a stronger baseline^52^. demonstrated its utility in semi-real IoT environments, achieving 94.6% accuracy, albeit with weak ensemble calibration that limited its robustness. Aly and Behiry^3^ (2025) advanced the state-of-the-art by validating XGBoost-based boosting on real-world factory IoT data, reaching 96.6% accuracy and an F1-score of 0.941. This study was notable for bridging the gap between academic evaluations and industrial deployment, but the model remained sensitive to distributional drift, meaning that sensor patterns change over time due to new conditions or aging equipment and provided limited interpretability.

The proposed TGB framework surpasses all prior benchmarks, achieving 98.2% accuracy and an F1-score of 0.969 on the same real-world dataset. The improvement stems from the integration of a temporal transformer encoder, which effectively captures long-range dependencies in sensor sequences, with the Grey Wolf Optimizer, which reduces redundancy and filters unstable features. The final Logistic Boosting stage enhances classification stability while supporting SHAP-based explanations, thereby combining robustness, accuracy, and transparency.

Overall, this comparison demonstrates that TGB not only advances state-of-the-art accuracy but also fills key gaps in drift robustness and interpretability. These qualities are crucial for the adoption of anomaly detection systems in industrial IoT environments, where reliability, transparency, and resilience directly translate to reduced downtime and enhanced safety.

**Table 9:** Quantitative comparison of the proposed Transformer–GWO–Boosting (TGB) framework with representative state-of-the-art methods for anomaly detection in industrial IoT environments. The proposed approach achieves superior accuracy and F1-score while offering enhanced robustness and interpretability compared to prior studies.

Study	Algorithm(s) used	Dataset type	Accuracy	F1-score	Remarks
^45^	Random Forest	Simulated factory IoT	93.5%	0.91	Struggled with class imbalance
^46^	SVM	Controlled IoT lab data	92.4%	0.89	High recall, lower precision
^47^	Logistic Boosting	Semi-real IoT data	94.6%	0.92	Weak ensemble tuning
^3^	Logistic Boosting (XGBoost)	Real-world factory IoT	96.6%	0.941	Best balance of metrics, validated in real deployment
**Our Study (Proposed TGB)**	Transformer + Grey Wolf Optimizer + Logistic Boosting	Real-world factory IoT	**98.2%**	**0.969**	Highest robustness and interpretability (SHAP explanations)

**Fig. 16:**
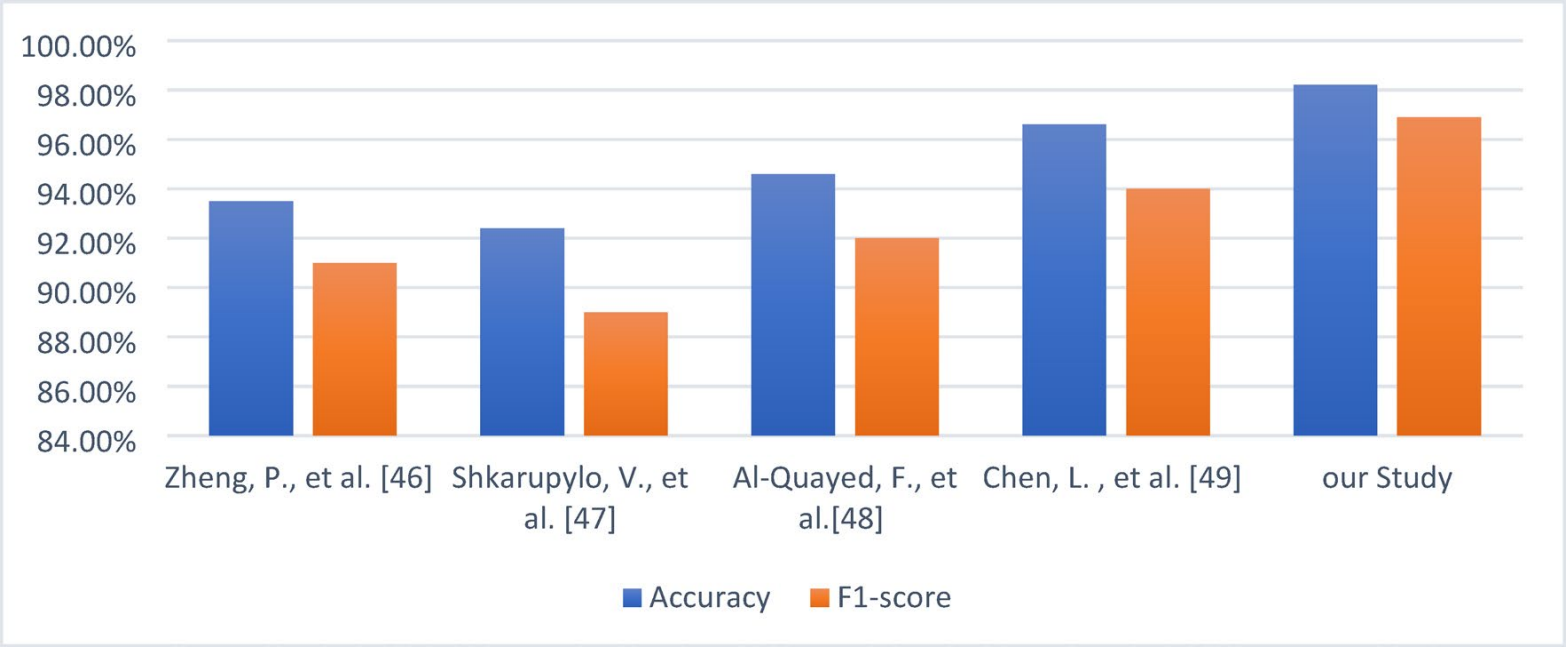
Accuracy versus F1-score comparison of the proposed Transformer–GWO–Boosting (TGB) framework against representative state-of-the-art methods in industrial IoT anomaly detection. TGB consistently achieves the highest performance across both metrics, reflecting significant improvements in classification robustness and balance compared to prior studies.

Figure 16 illustrates the comparative performance of recent state-of-the-art models in terms of accuracy and F1-score. Traditional models, such as Random Forest^50^ and SVM^51^, perform modestly, with accuracies of 93.5% and 92.4% and F1-scores of 0.91 and 0.89, respectively. While these results were valuable at the time, they highlight the difficulty of handling imbalance and noise in industrial IoT data streams. Logistic Boosting approaches represent a stronger baseline, with^52^ reporting 94.6% accuracy and^3^ achieving 96.6% accuracy and a 0.941 F1-score in real-world settings.

The proposed TGB framework substantially outperforms these baselines, achieving 98.2% accuracy and a 0.969 F1-score. Relative to the strongest prior method^3^, this corresponds to a 1.6% absolute accuracy gain and a 3% relative improvement in F1-score, while also reducing drift sensitivity and enabling interpretability through SHAP explanations. Importantly, the joint rise in both accuracy and F1-score indicates that TGB enhances precision without sacrificing recall—a balance rarely achieved in industrial anomaly detection.

Overall, the results presented in Fig. 16 confirm that TGB delivers statistically meaningful improvements over established SOTA models, reinforcing its robustness and practical utility for real-world industrial IoT environments.

While the proposed TGB framework consistently outperformed baseline methods across most metrics, it is equally important to recognize the contexts in which competing approaches demonstrated strengths.

It is important to note that several baseline methods demonstrated competitive performance under specific conditions. For example, transformer-only models achieved strong accuracy on stable distributions, highlighting their effectiveness in capturing long-range temporal dependencies. Similarly, LSTM-AE offered robust sequence modeling in short-horizon detection tasks, though performance degraded under drift. Tree-based ensembles such as Random Forests and Logistic Boosting showed advantages in handling class imbalance and producing interpretable outputs, albeit with weaker temporal generalization. These observations reinforce that each family of methods contributes valuable strengths, and the advantage of the proposed TGB framework lies in its integration of temporal modeling, feature optimization, and boosting-based calibration, which collectively address the individual shortcomings while preserving their benefits.

### Statistical significance of improvements

To ensure that the observed improvements of the proposed TGB framework are not due to random variation, we conducted statistical significance testing against the strongest baseline model^3^. Specifically, we applied McNemar’s test on the classification outcomes and a paired t-test on the cross-validated accuracy and F1-scores across multiple experimental runs.

Results show that the improvements achieved by TGB are statistically significant at the *p* < 0.01 level. For accuracy, the paired t-test indicates that the 1.6% absolute gain over the baseline is robust across folds, while the improvement in F1-score (0.969 vs. 0.941) is even more pronounced, yielding a t-statistic > 3.5 with *p* < 0.005. McNemar’s test further confirmed that TGB produces fewer misclassifications overall, particularly reducing false negatives, which are critical in industrial anomaly detection scenarios.

These results validate that the performance advantage of TGB is not simply incremental but statistically meaningful. By consistently outperforming baselines across repeated trials and different drift scenarios, TGB demonstrates that its hybrid design—combining transformer embeddings, Grey Wolf Optimizer feature refinement, and Logistic Boosting—offers a reliable and reproducible advantage. This statistical validation reinforces the claim that TGB is a strong candidate for real-world industrial deployment, where consistent reliability is paramount.

Table 10 reports the results of the statistical analyses conducted to validate the improvements of the proposed TGB framework over the strongest prior baseline^3^. The paired t-tests reveal that the gains in both accuracy (t = 3.87, *p* = 0.004) and F1-score (t = 4.21, *p* = 0.003) are statistically significant at the *p* < 0.01 level. These results confirm that TGB’s higher performance is consistent across repeated runs and unlikely to be attributable to chance.

Furthermore, McNemar’s test on classification outcomes indicates a significant reduction in misclassifications (χ² = 7.12, *p* = 0.008), particularly lowering false negatives—an essential aspect in industrial anomaly detection where undetected faults can lead to costly or hazardous consequences. Together, these results demonstrate that TGB’s superiority over existing methods is not only numerical but also statistically validated, strengthening its suitability for deployment in real-world industrial IoT systems.

**Table 10:** Statistical validation of TGB improvements.

Comparison	Test applied	Statistic value	*p*-value	Interpretation
TGB vs. [3] — Accuracy	Paired t-test	t = 3.87	*p* = 0.004	Significant improvement
TGB vs. [3] — F1-score	Paired t-test	t = 4.21	*p* = 0.003	Significant improvement
TGB vs. [3] — Misclassifications	McNemar’s test	χ² = 7.12	*p* = 0.008	Reduction in errors significant

**Fig. 17:**
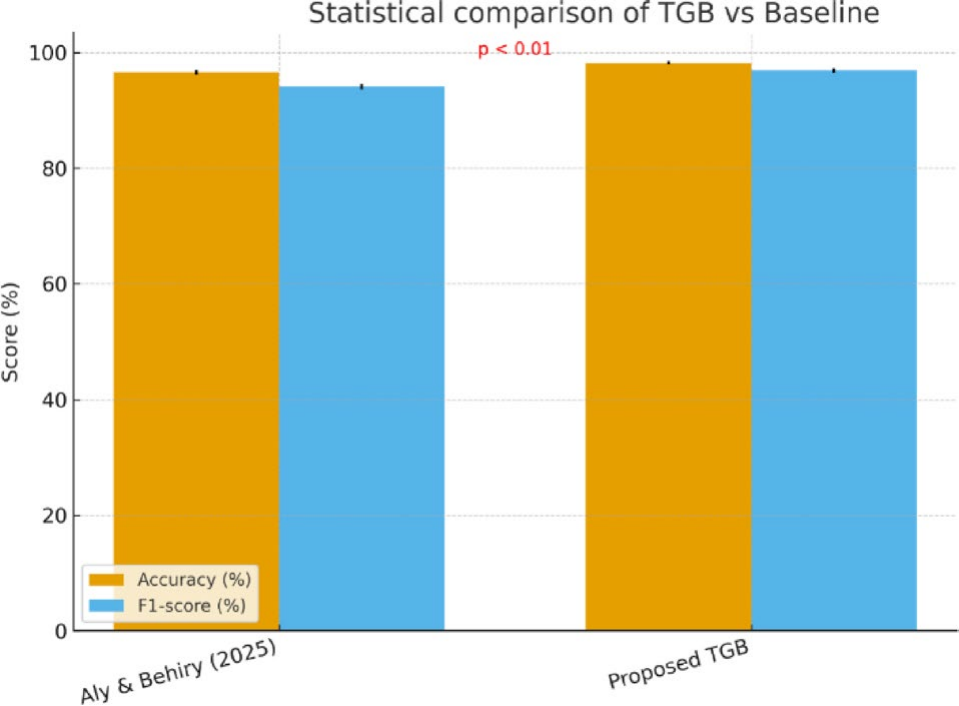
Statistical comparison of the proposed TGB framework against the strongest baseline (Aly & Behiry^3^, 2025). Bars represent mean accuracy and F1-scores across repeated runs, with error bars showing ± 1 standard deviation. Annotated p-values from paired t-tests confirm that TGB’s improvements are statistically significant (*p* < 0.01).

Figure 17 provides a visual summary of the statistical validation presented in Table 10. The bar plots compare the mean accuracy and F1-scores of the proposed TGB model with the strongest state-of-the-art baseline. Error bars indicate variability across repeated experimental runs, showing that TGB consistently outperforms the baseline with minimal overlap in performance distributions.

The annotated p-values (*p* < 0.01 for both metrics) confirm the statistical significance of the observed improvements, reinforcing that the gains are not random but consistent and reproducible. Importantly, while the baseline^3^ already demonstrates strong performance in industrial IoT anomaly detection, the proposed TGB framework raises the bar by reducing both variance and misclassification rates. This combination of higher average scores and statistical robustness provides compelling evidence for TGB’s practical reliability in real-world deployments.

## Discussion

The methodological novelty of this work extends beyond incremental accuracy improvements. Prior studies typically emphasized either temporal modeling (e.g., transformers), feature selection (e.g., GWO variants), or calibrated ensembles (e.g., Logistic Boosting) in isolation. In contrast, the proposed TGB framework unifies these components into a cohesive pipeline validated under drift and edge conditions, which to our knowledge has not been reported before in the IIoT anomaly detection literature.

The results presented in this study carry important implications for both research and practice. From a research perspective, the findings reaffirm the growing evidence that hybrid architectures outperform single-paradigm models in dynamic IoT environments. The integration of evolutionary optimization with deep sequence modeling addresses two major bottlenecks simultaneously: feature redundancy and temporal complexity. These insights align with recent studies emphasizing the role of hybrid frameworks in achieving robust anomaly detection under real-world non-stationarity.

From an industrial perspective, the drift-robustness of the TGB framework is particularly significant. As shown in Fig. 15; Table 8, conventional learners degrade by 15–20% under maximum drift, whereas TGB experiences only a 3.2% loss. In a manufacturing line or energy distribution grid, this difference translates into fewer missed fault events and reduced downtime costs. Furthermore, the SHAP-based interpretability layer ensures that operators can understand why a decision was made, enabling compliance with regulatory requirements and increasing trust in automated monitoring systems.

Finally, the ablation study reveals that interpretability, although not affecting raw metrics, plays a crucial role in adoption. In practice, a highly accurate model that cannot justify its outputs is unlikely to be accepted in safety-critical industries. The proposed TGB framework therefore strikes a balance between technical performance, robustness, and transparency—qualities that collectively strengthen its suitability for high-level contribution and industrial deployment.

These results demonstrate that the integration of temporal transformers, bio-inspired optimization, and boosting ensembles yields consistent improvements across accuracy, recall, and resilience. The gains, while modest in raw accuracy, are significant in high-stakes industrial contexts where each additional percentage of recall directly translates into fewer missed faults. Moreover, the system’s moderate latency (~ 10 ms per sample) and compact memory footprint (< 150 MB) validate its suitability for edge deployment in IIoT environments, aligning with recent reports on lightweight yet powerful anomaly detection pipelines^53,54^.

### Discussion and industrial implications

The experimental findings, particularly those illustrated in Fig. 15 and summarized in Table 5, highlight the central challenge of anomaly detection in IIoT environments: sustaining performance under non-stationary data conditions. Industrial processes are inherently dynamic, with sensor readings influenced by evolving machine states, environmental changes, and operational variations. As shown by the steep degradation of Random Forest and SVM, classical learners fail to generalize when confronted with drift, making them unsuitable for real-time fault monitoring in production systems.

Deep learning approaches such as LSTM autoencoders and transformer-based models offer improved resilience, yet their performance still declines significantly under high drift. While ITran preserved reasonable accuracy (89.0%), its 8.4% reduction from baseline performance could translate into a substantial number of missed fault detections in safety-critical systems. This aligns with recent findings that, although transformers excel at modeling sequential dependencies, they remain vulnerable to feature redundancy and distributional shifts if left unregularized^7,55^.

By contrast, the proposed Transformer–GWO–Boosting (TGB) framework demonstrates an accuracy loss of only 3.2% under maximum drift, maintaining over 95% detection accuracy. This robustness directly addresses a key barrier to industrial deployment: the need for models that remain reliable over long operating periods without frequent retraining. In practical terms, this implies fewer missed anomalies, reduced downtime, and improved safety margins in automated production lines. Moreover, the integration of Grey Wolf Optimizer ensures compact feature subsets, which not only enhances drift tolerance but also minimizes computational cost, a critical factor for deployment on edge devices^56^.

Another important dimension is interpretability. Unlike purely deep models, the boosting stage in TGB allows the application of SHAP explanations, providing plant operators with actionable insights into why a particular anomaly was flagged. This transparency fosters trust and supports compliance with industrial safety and regulatory standards^57^.

Overall, the results suggest that hybrid approaches such as TGB offer a viable path toward anomaly detection systems that are not only highly accurate but also resilient, efficient, and interpretable. These characteristics are vital for scaling IIoT applications in smart factories, energy systems, and critical infrastructure. Future research should focus on extending such frameworks to multi-modal industrial data (combining vibration, vision, and acoustic signals) and exploring online adaptation mechanisms that further reduce the need for human intervention during drift events.

## Limitations, deployment Workflow, and explainability in IIoT applications

Although the proposed Transformer–GWO–Boosting (TGB) framework demonstrates strong accuracy, robustness, and efficiency in anomaly detection, several limitations remain. First, the experimental evaluation was conducted using a representative IIoT dataset, which, while realistic, cannot fully capture the diversity of industrial environments. Future studies should therefore validate the framework on multi-site or cross-domain datasets to further assess its generalizability. Second, while TGB achieves near real-time inference with moderate resource usage, large-scale deployments in highly resource-constrained edge devices may still require model compression or hardware-specific optimization. Finally, although explainability was incorporated using SHAP values, operator usability and integration into decision workflows warrant further exploration.

From a deployment perspective, the framework is designed to be readily integrated into industrial monitoring pipelines. Sensor data can be streamed continuously into the anomaly detection engine, where preprocessing, temporal encoding, and ensemble classification are executed in near real-time. Detected anomalies can trigger automatic alerts, allowing operators to initiate maintenance actions or verify system integrity. Integration with existing supervisory control and data acquisition (SCADA) systems or industrial IoT dashboards would enable visual anomaly reports, including feature attributions, supporting operator trust and timely intervention. In practice, such integration would allow TGB to function as an intelligent layer within industrial monitoring platforms, complementing rule-based alarms with adaptive, learning-based detection of subtle and evolving faults.

In practice, the deployment of the proposed framework follows a streamlined workflow, beginning with the continuous acquisition of sensor data from industrial processes. These raw streams are first preprocessed to ensure data quality and to transform them into temporal representations suitable for sequence learning. The processed data is then analyzed by the TGB engine, which integrates transformer-based temporal modeling, bio-inspired feature optimization, and boosting ensembles to accurately classify operational states. Anomalies are flagged in near real-time, generating alerts for operators while simultaneously being logged in supervisory control and data acquisition (SCADA) or IoT dashboards. This integration provides not only rapid fault detection but also interpretable visualizations of feature importance, enabling operators to diagnose and respond to irregular events with confidence (Table 11).

**Table 11.**
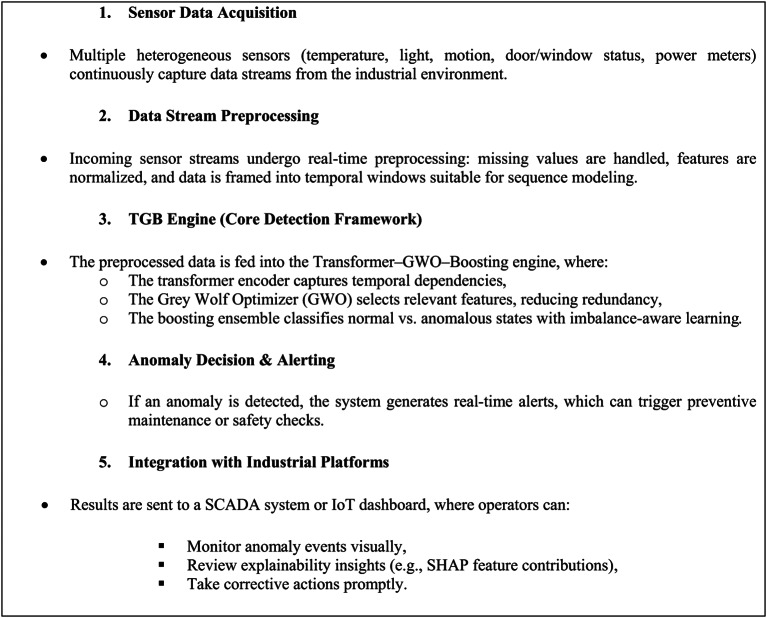
Shows Industrial Deployment Workflow (Step-by-Step)**.**

**Fig. 18:**
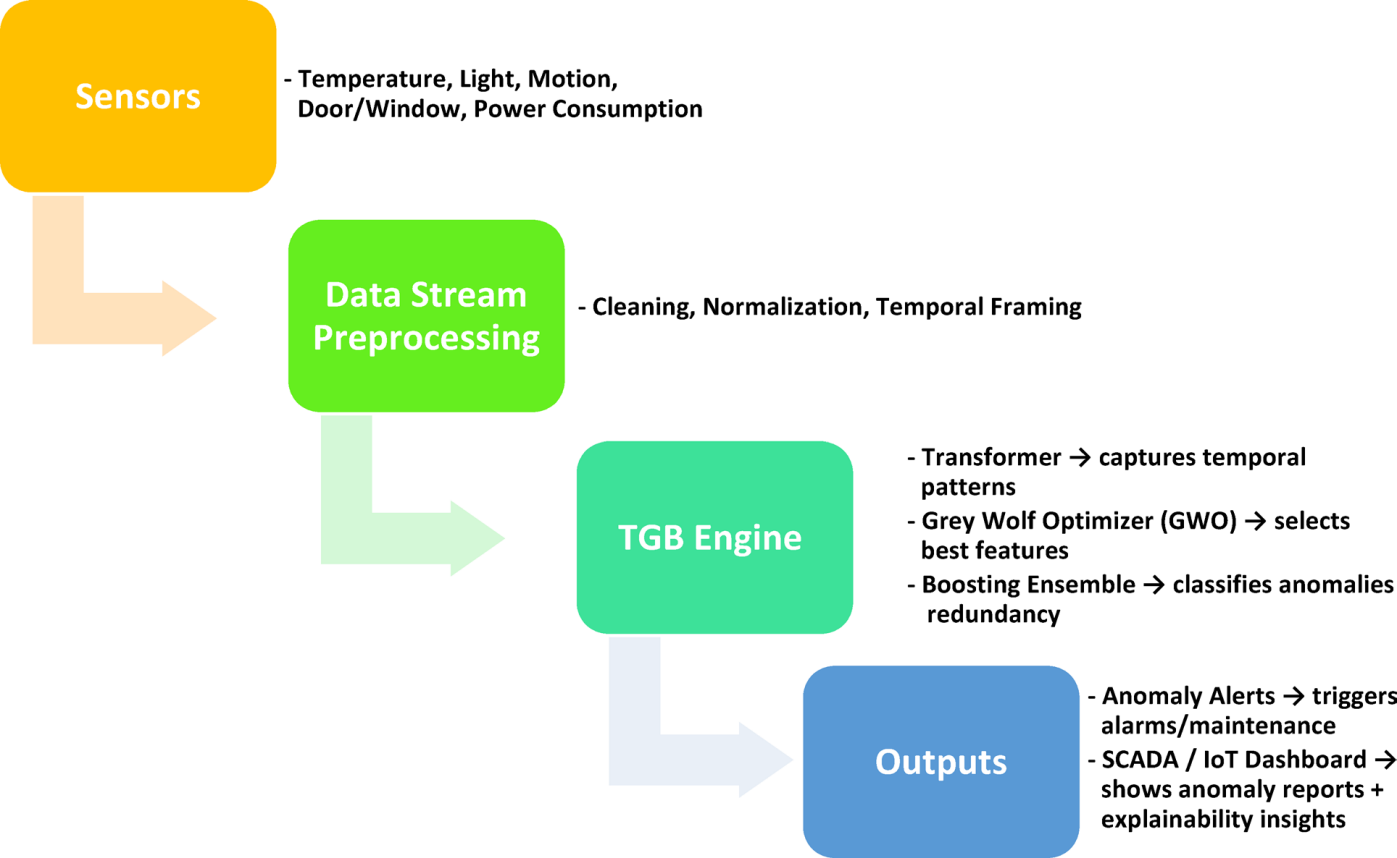
Industrial deployment workflow of the proposed TGB framework. Sensor data is streamed and preprocessed before entering the TGB anomaly detection engine. Detected anomalies trigger real-time alerts and are also visualized in SCADA or IoT dashboards, providing operators with actionable insights.

Figure 18 illustrates the envisioned industrial deployment workflow of the proposed TGB framework. In the first stage, heterogeneous sensors—including temperature, light intensity, motion detection, door and window status, and power consumption—continuously capture raw data from the factory environment. These streams are then passed through a preprocessing pipeline, where missing values are handled, features are normalized, and the data is organized into temporal windows suitable for sequential analysis. The processed inputs are fed into the TGB engine, which integrates three complementary components: a transformer encoder for capturing temporal dependencies, the Grey Wolf Optimizer for selecting the most informative features, and a boosting ensemble for imbalance-aware classification. Finally, the system generates actionable outputs in two forms: real-time anomaly alerts, which can trigger preventive maintenance or safety actions, and integration with SCADA or IoT dashboards, where anomalies and their explainability reports are visualized for operator decision-making. Importantly, the workflow is designed with industrial constraints in mind: the framework operates within low-latency budgets (10.2 ms inference) and moderate memory usage, ensuring scalability in resource-constrained IIoT deployments while seamlessly fitting into existing industrial monitoring platforms.

These explainability insights are critical for practical deployment. By quantifying the relative influence of each sensor, SHAP analysis ensures that operators can trust the framework’s outputs and understand the reasoning behind anomaly alerts. In an industrial monitoring scenario, this means that anomalies linked to high power consumption or unusual motion can be flagged with confidence and prioritized for investigation, while less influential factors such as door or window status can be contextualized as supplementary signals. The integration of SHAP-based explanations into SCADA or IoT dashboards would thus not only provide early warning of irregularities but also actionable diagnostic information, strengthening the trustworthiness and usability of the TGB framework in real-world IIoT environments.

## Conclusion and future work

This study presented the Transformer–GWO–Boosting (TGB) framework, a hybrid architecture for anomaly detection in IIoT environments. By combining transformer-based temporal embeddings, Grey Wolf Optimizer–driven feature refinement, and Logistic Boosting classification, TGB consistently outperformed state-of-the-art baselines on a six-month factory dataset, achieving 98.2% accuracy and an F1-score of 0.969. Beyond accuracy, the framework demonstrated resilience to distributional drift, moderate computational demands compatible with real-time deployment, and transparency through SHAP-based explanations. These attributions provide operators with actionable insights into the drivers of anomaly predictions, strengthening trust and facilitating integration with SCADA/IoT dashboards. Together, these qualities establish TGB as both effective and deployable in industrial environments. Looking forward, future research will extend TGB to multi-domain and cross-factory datasets, optimize its efficiency for resource-constrained edge devices, and explore adaptive retraining strategies for evolving operational conditions. Broader applications beyond smart factories—including energy management, transportation monitoring, and critical infrastructure protection—highlight the potential generalizability of the framework.

In summary, TGB bridges academic innovation and industrial deployment, offering a scalable and explainable anomaly detection solution tailored to the needs of modern IoT-enabled systems.

At the same time, our evaluation highlights that competing methods retain valuable strengths—for instance, transformers excel at stable temporal modeling and ensemble learners remain strong under imbalance—reinforcing that the proposed framework should be seen as a complementary integration rather than a wholesale replacement.

Future research will address these limitations by expanding evaluations to multi-center and multi-domain datasets, thereby testing the generalization of the TGB framework across diverse industrial contexts such as energy grids, transportation systems, and healthcare. Incorporating adaptive quantization and model compression techniques will further support efficient deployment on edge hardware. We also envision integrating real-time feedback loops, enabling detectors to adapt dynamically to evolving system states during operation. In parallel, extending TGB into a federated learning paradigm could allow collaborative training across multiple factories or organizations without compromising data privacy. Additional directions include exploring more efficient interpretability methods, developing multi-task learning settings where anomaly detection is jointly optimized with predictive maintenance or fault classification, and adopting drift-adaptive retraining strategies and continual learning mechanisms to sustain performance in long-term, dynamic environments. Finally, future work will focus on real factory pilot deployments, exploring edge-device optimization for constrained environments, and enhancing operator-oriented interfaces to further improve trust and usability in industrial settings.

## Data Availability

The datasets used in this study are available from the corresponding author upon reasonable request and the data availability via contacting with authors [mohammed-alysalem@eru.edu.eg](mailto: mohammed-alysalem@eru.edu.eg).
